# Recent Advances on the Analysis and Biological Functions of Cinnamaldehyde and Its Derivatives

**DOI:** 10.3390/antiox14070765

**Published:** 2025-06-22

**Authors:** Roghayeh Karimirad, Baskaran Stephen Inbaraj, Bing-Huei Chen

**Affiliations:** 1 Department of Food Science, Fu Jen Catholic University, New Taipei City 242062, Taiwan; karimirad2020@gmail.com (R.K.); sinbaraj@yahoo.com (B.S.I.); 2 Department of Nutrition, China Medical University, Taichung 404328, Taiwan

**Keywords:** cinnamon, cinnamaldehyde, analytical methods, pharmacokinetics, chronic disease prevention

## Abstract

Natural antioxidants isolated from fruits, vegetables, herbs and spices have drawn great attention owing to their numerous health-promoting effects. Cinnamaldehyde (CA), an abundant antioxidant in cinnamon spice, has been explored more intensely over the last decade as it has been demonstrated to be effective and safe in the treatment of various diseases. Structurally, a substituted aldehyde group with an unsaturated carbon–carbon double bond with two electrophilic sites for reaction with receptors and enzymes can exert diverse biological effects. Although cinnamon has been traditionally used as a spice and herbal remedy, many studies investigating the most dominant functional compound, CA, and its biological activities have been reported in recent years. This review article intends to present an overview of recent advances in analytical methods and the application of cinnamon extract/oil, CA and its derivatives, CA-polymer/biomolecule conjugates and CA micro/nanosystems in alleviating various chronic diseases including cancer, diabetes, obesity, cardiovascular disease, neurological disorders, osteoarthritis and osteoporosis. Both in vitro and in vivo studies have demonstrated the improved pharmacological efficiency of CA and its derivatives as well as their polymer/drug/biomolecule conjugates and micro/nanoencapsulated forms, suggesting a possible alternative natural therapy and adjuvant therapy with conventional drugs via a synergistic process.

## 1. Introduction

Cinnamon, a popular spice with rich flavor and aroma, can be obtained from several plant species of the genus *Cinnamomum*, belonging to the taxonomy division *Magnoliophyta*, order *Magnoliales*, class *Magnoliopsida* and family *Lauraceae* [1]. It is an evergreen tree that grows up to 10–15 m in height, and approximately 250 *Cinnamomum* species have been identified all around the world, especially in China, India, Sri Lanka, Taiwan, Vietnam and Indonesia [2,3]. Cinnamon contains many bioactive constituents including cinnamaldehyde, cinnamic acids, phenolic acids, flavonoids, volatile oil, flavor compounds, terpenoids, lignans, saponins, polysaccharides, etc., and thus is traditionally used as a food additive as well as in medicinal formulations for the treatment of common ailments [4,5]. Among various bioactive compounds, cinnamaldehyde (CA) has been identified as the most abundant and important antioxidant responsible for various pharmacological functions, with its content varying depending on the species, growth location and environment [6,7,8,9,10]. The essential oil isolated from different cinnamon species was reported to contain 85.3%, 90.5%, 92.0%, 79.8%, 69.6%, 65.6%, 73.6%, 56.1% and 46.6% of CA in *C. cassia*, *C. verum* (*C. zeylancium*), *C. burmanii*, *C. osmophloeum*, *C. loureirri*, *C. sulphuratum*, *C. Tamala*, *C. pubescens* and *C. aureofulvum*, respectively [1,3]. Although *C. cassia* and *C. verum* are the two most common species, they contain high levels of coumarin, a toxic compound demonstrated to be hepatotoxic and carcinogenic [4]. However, the native cinnamon species of Taiwan *C. osmophloeum* was reported to contain only trace amounts of coumarin, and thus should possess greater safety and economic value than some other cinnamon species [11].

CA (3-phenyl-2-propenal) is a yellowish oily liquid with a pleasant odor and taste. It was first isolated from cinnamon oil in 1834 by Andre Dumas and Peligot, but later synthesized in 1854 in a laboratory by Chiozza [1]. Structurally, it has a molecular formula of C_9_H_8_O consisting of a phenyl ring with a substituted aldehyde group possessing an unsaturated carbon–carbon double bond. CA has a molecular mass of 132.16 g/mol with moderate solubility in water (1.1 g/L at 20 °C) and higher solubility in organic solvents including ethanol and ether, with density at 1.05 g/mL (25 °C), boiling point at 248 °C and melting point at −7.5 °C [12]. Furthermore, it has several other characteristics, including molecular surface area at 194.04 Å^3^, polar surface area at 17.07 Å^2^, polarizability at 15.78, molar refractivity at 42.13, pKa value at −4.44, and particle coefficient (log P) at 1.98, with one hydrogen bond acceptor and without any hydrogen bond donor, satisfying the popular Lipinski rule of thumb for an orally active drug with improved pharmacokinetics in the human body [1]. CA is generally regarded as safe (GRAS) by the United States Food and Drug Administration (USFDA) and Flavor/Extract Manufacturer’s Association (FEMA) as a flavor ingredient, while the Council of Europe has grouped CA into the list of substances with ‘A’ status, meaning ‘may be used in foodstuffs’ [13,14]. Several toxicological studies have also confirmed CA to be safe without exerting any genotoxic or carcinogenic effects, with the highest exposure level being 4100 ppm [15,16].

The structure–activity relationship of CA and its derivatives is mainly related to their ability to act as electrophiles for reaction with certain receptors and enzymes exerting diverse pharmacological functions [14]. Specifically, the two electrophilic reactive sites include the β-carbon on the conjugated double bond and the carbon of the aldehyde carbonyl group. Several studies have associated the bioactivity of CA and its derivatives with the nucleophilic attack of unsaturated β-carbon (Michael acceptor ability). For example, Cabello et al. [17] demonstrated that CA and 4-methoxycinnamaldehyde with intact Michael acceptor ability were able to inhibit the proliferation of human metastatic melanoma cell (A375) with IC_50_ at <10 μM by upregulating heme oxygenase-1, sulfiredoxin-1, thioredoxin reductase-1 and cyclin-dependent kinase inhibitor-1A, as well as inhibiting nuclear factor kappa B (NF-κB) and tumor necrosis factor-alpha (TNF-α)-induced interleukin (IL)-8 production, thereby arresting the cell cycle at G1. For the same reason, naturally occurring CA derivatives such as 2-hydroxycinnamaldehyde and 2-benzoyloxycinnamaldehyde have been shown to exert anti-inflammatory and anticancer activities [18]. On the other hand, the presence of unsaturated carbonyl carbon in CA and its derivatives has been shown to exhibit antimutagenic activity, with some studies also concluding that carbonyl carbon is more significant than β-carbon in terms of reactivity [14].

Owing to the abundance of CA in cinnamon, it has traditionally been used for centuries as both a spice and a herbal remedy [14]. Most importantly, based on Chinese philosophy, cinnamon helps to regulate the human’s Yang energy through the restoration and flow of energy, as well as alleviating cold and discomfort [6]. Additionally, CA has been widely incorporated into medicinal formulations for the attenuation of various health conditions, including respiratory and kidney problems, back and knee pain, dizziness and eye redness, heart and abdomen discomfort, diarrhea, pain during menstruation, erectile dysfunction, hernia, etc. [6]. Furthermore, there has been an upsurge in the exploration of various pharmacological functions of CA and its derivatives, with many studies demonstrating their physiological release, pharmacokinetics and bioavailability [19,20], antioxidant and anti-inflammation effects [21,22], antimicrobial property [1,14], anticancer [23,24] and antidiabetic activity [4,25], anti-obesity effects [26,27], cardiovascular disease protection [28,29], neuroprotection [5,30] and bone health improvement effects [31,32]. Studies focusing on developing green and sustainable extraction methods without compromising extraction efficiency, as well as advanced analytical methods for the rapid, robust and highly sensitive analysis of CA and other bioactive components of cinnamon, have also been reported [4,5].

Furthermore, as essential oils from various plant sources have been explored for their application in food packaging and preservation [33,34], many studies have demonstrated that the incorporation of cinnamon essential oil and CA into biopolymer-, metal nanoparticle- and nanoemulsion-based food packaging films/coatings could enhance their physicochemical, antimicrobial, antioxidant and sensory properties, aiding in the efficient preservation and extension of the shelf-life of fruits, vegetables, fish and meat products [35,36,37,38,39,40,41]. Specifically, several physicochemical properties such as thickness, color index, transparency, water content, water solubility, water contact angle, mechanical and water barrier were shown to be improved in CA based films/coatings [41]. Both cinnamon essential oil and CA were shown to exert a similar antibacterial efficiency through the inhibition of cell division, the suppression of adenosine triphosphatase and the modification of the cell membrane, resulting in bacterial cell death [35]. For instance, cinnamon oil was shown to exhibit a minimum inhibitory concentration (MIC) ranging from 0.052 to 0.178 mg/mL, as well as a minimum bactericidal concentration (MBC) of 0.058–1.084 mg/mL, against *Listeria monocytogenes*, *Staphylococcus aureus*, *Escherichia coli* and *Salmonella typhimurium* [42]. In several different studies, CA was reported to be effective against 12 Gram-positive and Gram-negative bacteria with MIC values ranging from 0.160 to 0.630 mg/mL [43]. Likewise, cinnamon essential oil was reported to exhibit antifungal effects against various fungi and yeasts by disrupting the cell wall and membrane, coagulating the cytoplasm, and subsequently damaging cellular organelles, resulting in the leakage of cell contents [44]. Cinnamon oil and CA were also shown to act as antioxidants by preventing lipid oxidation for the preservation of freshness and nutritional quality of foods and the extension of shelf life [35]. However, the application of cinnamon essential oil and CA in food packaging and preservation will be reviewed in a separate review article in the future.

Therefore, this review article aims to provide an overview of recent advances on the analysis methods and pharmacological functions of cinnamon extract, CA, and its derivatives.

## 2. Biosynthesis of Cinnamaldehyde and Its Derivatives

**Figure 1:**
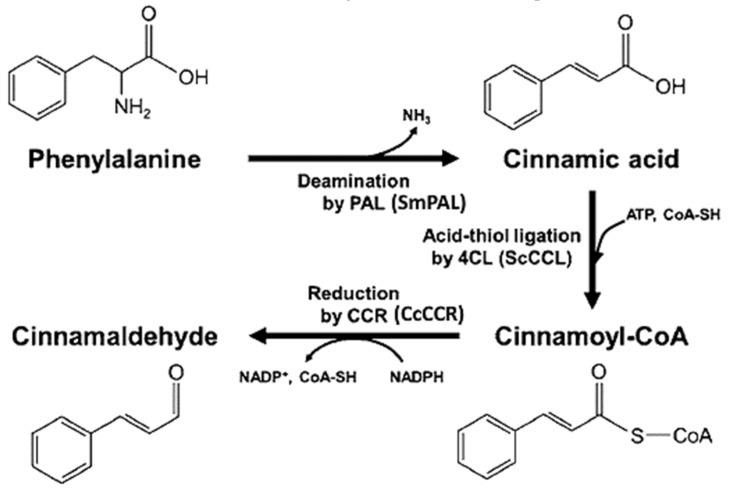
Biosynthesis of cinnamaldehyde.

CA can be synthesized from L-phenylalanine by three sequential enzymatic reactions involving (1) the non-oxidative deamination of L-phenylalanine to form cinnamic acid using phenylalanine-ammonia lyase (PAL), (2) the conversion of cinnamic acid to cinnamoyl-coenzyme A (CoA) by the acid-thiol ligation reaction catalyzed by 4-coumarate-CoA ligase (4CL) using adenosine triphosphate (ATP), and (3) the reduction of cinnamoyl-CoA to cinnamaldehyde by cinnamoyl-CoA reductase (CCR) using nicotinamide adenine dinucleotide phosphate (NADPH) (Figure 1) [13,45]. Bang et al. [45] evaluated two PAL enzymes, taking one from the plant *Arabidopsis thaliana* (AtPAL) and the other from the bacterium *Streptomyces maritimus* (SmPAL) for the comparison of efficiency in producing cinnamic acid from phenylalanine, and found that the latter exhibited 21-fold and 27-fold higher activity than the former at 30 °C and 37 °C, respectively. Likewise, the 4CL enzyme from *S. coelicolor* (ScCCL) was shown to exert higher activity than that from *A. thaliana* (At4CL) for the conversion of cinnamic acid to cinnamoyl-CoA, while the CCR enzyme from *A. thaliana* (AtCCR1) was used for the effective conversion of cinnamoyl-CoA to CA [45]. However, by using the three enzymes SmPAL, ScCCL and AtCCR1, a lower CA yield (75 mg/L) was derived, which is a limiting factor as regards its commercial production. To overcome this issue, Ye et al. [46] demonstrated that a CCR enzyme cloned from *C. cassia* (CcCCR1) could exhibit 14.7-fold higher activity than AtCCR1 in converting cinnamoyl CoA to CA, suggesting that SmPAL, ScCCL and CcCCR1 can be utilized for the large-scale commercial production of CA.

CA derivatives can be synthesized by several methods including aldol condensation, the Wittig reaction, the Heck/oxidative Heck reaction, and allylic alcohol oxidation [47,48,49]. Aldol condensation typically involves the condensation of two carbonyl compounds, i.e., between an aldehyde and aldehyde or ketone, through nucleophilic reaction in the presence of a base to form conjugated enones. In a study dealing with the antifungal activity of CA on green mold, Gan et al. [47] synthesized various CA derivatives including 4-chloro CA, 4-bromo CA, 4-nitro CA, 4-methyl CA, 4-methoxy CA and 2,4-dimethoxy CA by refluxing a 1:1:1 mixture of benzaldehyde, acetaldehyde and NaOH, followed by cooling and extracting with ethyl acetate and evaporating to dryness for the further purification of crude extract by column chromatography.

**Figure 2:**
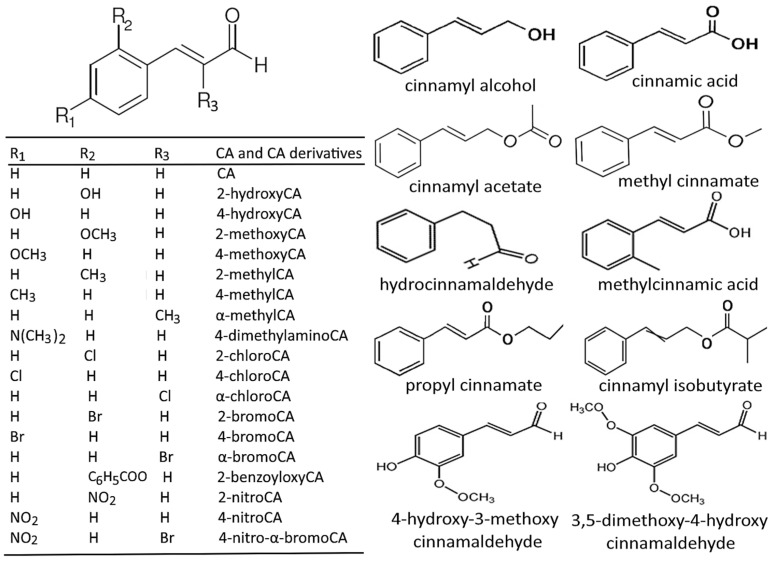
Chemical structures of CA and some biologically active derivatives of CA. CA, cinnamaldehyde.

The Wittig reaction is the most widely used method for the synthesis of CA derivatives and involves the reaction of an aldehyde or ketone with a stabilized phosphonium ylide to form an alkene, while, in the modified Wittig reaction, a selective formation of (E)-alkenes is made possible through the incorporation of excess lithium salts. By using the Wittig reaction, Doyle et al. [48] synthesized a total of 15 CA derivatives for the evaluation of antibacterial activity. For the synthesis of CA derivatives by the Heck reaction, an alkene or alkyne is coupled with an aryl or vinyl halide in the presence of palladium catalyst, with oxidative Heck reaction being performed under mild conditions without involving elevated temperature or base. Nordqvist et al. [49] employed a base-free oxidative Heck reaction for the synthesis of α,β-unsaturated CA derivatives, which were used as precursors for the further synthesis of novel α-aryl-substituted fosmidomycin analogues to evaluate their inhibition effects on *Mycobacterium tuberculosis* infection. Moreover, allylic alcohols can be oxidized to CA derivatives by using various oxidizing agents in the allylic alcohol oxidation method [49]. Some other methods for the synthesis of CA derivatives include the Horner–Wadsworth–Emmons reaction, the Peterson reaction, and the reduction of carboxylic acid derivatives [49]. Figure 2 shows the chemical structures of CA and some biologically active derivatives of CA.

## 3. Methods for Cinnamaldehyde Analysis

### 3.1. Extraction

Extraction and purification are routinely used for isolating natural compounds from plant sources. The conventional methods for the extraction of bioactive compounds include hydrodistillation, decoction, the soxhlet extraction, steam distillation, maceration, digestion, reflux boiling, infusion, and percolation [50]. However, these methods suffer from many drawbacks such as high costs, a time-consuming nature, the consumption of a large amount of organic solvents, and poor solvent disposal and recycling practices, with a low yield due to thermal degradation. Recently, some more techniques have been developed to increase the quality and yield of bioactive compounds through the reduction of solvents, time length and processing cost. These techniques include microwave-assisted extraction (MAE), ultrasonic-assisted extraction (UAE), subcritical fluid extraction (SFE), supercritical fluid extraction (SCFE), enzyme-assisted extraction (EAE), and pulsed electric field extraction (PEFE) [51]. In comparison to conventional methods, the green techniques possess advantages such as lower temperature, shorter extraction time, less solvent consumption, a higher purity of target compounds, and eco-friendliness [50].

MAE is a technique for extracting analytes from the sample matrix into a solvent by utilizing microwave energy, but some of the non-volatile constituents have low solubility, resulting in a loss of non-volatile constituents in the extract. Recently, a new microwave-assisted extraction technique named microwave-assisted steam distillation (MASD) was introduced to overcome the drawback of the MAEs method. During the MASD extraction procedure, water is heated and converted into steam, which subsequently diffuses through the plant materials to extract the volatile components from the inner plant tissues. Due to the absence of water throughout the extraction procedure, the residue remains almost dry, facilitating the easy separation of non-volatile components. In this way, the plant materials can be separated from the water, preventing the loss of non-volatile components during extraction. Furthermore, this technique has been used in the extraction of *C. cassia*, where the extract was shown to contain CA (67.211%), 2-methoxycinnamaldehyde (15.9%), cinnamyl acetate (9.553%), 17 terpenes (2.724%), seven alcohols (2.325%), benzaldehyde (0.536%), o-anisaldehyde (0.531%), phenylethyl acetate (0.460%), benzyl benzoate (0.152%), and other aromatics (≤0.3%) [52].

Under the UAE technique, mechanical properties change the matrix surfaces of natural compounds, which enhances solvent penetration into inner structure of samples and allows for the dissolution of favorable constituents in the solution. The advantages of this technique are the short extraction time, moderate capital cost, ease of operation, and low level of consumption of solvent. A recent study has shown that a combination of UAE with hydrodistillation in the extraction of *C. cassia* resulted in a higher yield than that with hydrodistillation without ultrasound pre-treatment [53]. In another study, Yu et al. [52] compared three different extraction methods, including ultrasonic-assisted steam distillation (UASD), steam distillation (SDT), and microwave-assisted steam distillation (MASD), with a higher yield shown in UASD (8.33%) compared to that in SDT (3.91%). A total of 36 volatile compounds including aldehydes, alcohols, esters, aromatics, terpenes and ketones were present in cinnamon oil regardless of the extraction method employed, but a higher level of total CA was found for UASD.

In recent years, both SCFE and SFE techniques have been developed, with the latter representing a pressurized low-polarity fluid extraction method, and the former a green extraction method using supercritical fluid as an extraction solvent. Guo et al. [54] extracted cinnamon bark oil using the ultrasound-SFE method with temperature at 140 °C, pressure at 5 MPa, ultrasonic power at 145 W, ultrasonic frequency at 18.5 KHz and extraction time at 25 min; maximum yields of cinnamon oil and *E*-cinnamaldehyde of 1.78% and 12.662 mg/g, respectively, were obtained. Compared to conventional methods, SFE accelerated the extraction procedure, improved the quality of the extracted material, and decreased the usage of solvent. Nevertheless, compared to EAE, both methods are cheaper. For EAE, there are several drawbacks associated with industrial scale-up, including the expensive purification process, the incomplete decomposition of the plant cell wall and the restriction of enzyme usage under eco-friendly conditions [55].

For the PEFE method, a high or short-voltage electric field can generate holes in the cell walls of natural compounds, resulting in a better delivery of cellular components to enhance extraction efficiency. Pashazadeh et al. [51] reported that a yield of 5.06% was acquired when using the PEFE method by mixing 10 g of cinnamon powder and ethanol at a ratio of 1:10 (*w*/*v*), with pulse frequency at 1 Hz, pulse number at 40 and optimum voltage at 5.12 kV/cm. A rise in voltage to 4 kV/cm was shown to enhance the extraction yield of cinnamon, but this may encounter difficulties when applied in the industry because of high capital costs. Additionally, it requires a high-power treatment chamber with a special design and generator.

Collectively, these techniques require further investigation in terms of capital cost, extraction efficiency and possible industrial-scale production. In addition, the development of an optimum green extraction technology is necessary to enhance the extraction efficiency of bioactive compounds from cinnamon.

### 3.2. Analytical Methods for CA Analysis

Table 1 summarizes some recent analytical methods used for the determination of CA and other compounds in cinnamon bark and leaf.

#### 3.2.1. HPTLC Method

An alternative to high-performance liquid chromatography (HPLC) for CA determination is high-performance thin layer chromatography (HPTLC). Foudah et al. [56] developed an HPTLC method for the determination of CA in *C. burmannii*, *C. zeylanicum* and *C. cassia* via two extraction methods, ultrasound-based and traditional extractions. CA separation was carried out on glass-backed plates (10 × 20 cm) pre-coated with normal phase (NP)-18 silica gel 60 F254S, with a mobile phase of cyclohexane/ethyl acetate (90:10, *v*/*v*) in a linear ascending mode and detection wavelength at 296 nm. The CA contents were higher when using the ultrasound-based method than when using the traditional method. Based on the limit of detection (LOD) (3.56 ng/band), limit of quantitation (LOQ) (10.68 ng/band) and analytical greenness metric approach (AGREE) score (0.75), the HPTLC method was highly sensitive for CA quantitation. HPTLC is easy to carry out; however, the resolution of CA and its derivatives by HPTLC may be inadequate.

#### 3.2.2. HPLC Method

One of the major methods used for CA separation, identification and quantitation is HPLC. Puspita et al. [57] determined the CA content in cinnamon extract (*C. burmannii*) by mixing methanol and powdered cinnamon for sonication at room temperature, followed by separation with a C18 column (29 °C) and an isocratic mobile phase of acetonitrile and acetic acid (0.04%) at a ratio of 60:40 (*v*/*v*), detection wavelength at 280 nm and flow rate at 1.0 mL/min. A high recovery of 98.74–101.95% was shown, with the LOD and LOQ being 0.069 ppm and 0.23 ppm, respectively, and the CA content in cinnamon extract being 0.193%. Othman et al. [58] evaluated the optimal condition for CA extraction and separation by reversed-phase HPLC (RP-HPLC) with an isocratic mobile phase of water and acetonitrile at a ratio of 40:60 (*v*/*v*), column temperature at 40 °C, flow rate at 0.8 mL/min and detection wavelength at 280 nm. A maximum CA yield of 3.05 mg/g was obtained using an accelerated solvent extraction method with temperature controlled at 37 °C for 5 h.

In addition to HPLC, some more advanced techniques such as ultra-performance liquid chromatography–tandem mass spectrometry (UPLC-MS/MS) were used for CA analysis. A recent study identified 15 compounds in *C. osmophloeum* leaves by mixing ethanol (30% or 80%) and cinnamon powder for sonication at 60 °C for 2 h, followed by using a C18 100 Å LC column (1.6 μm particle size, 100 × 2.1 mm internal diameter (ID)) with an injection temperature of 30 °C and flow rate of 0.3 mL/min by employing a gradient mobile phase of acetic acid in water (A) and 0.025% acetic acid in methanol (B), and detection in multiple reaction monitoring (MRM) mode by UPLC-MS/MS [4]. Additionally, a yield of CA that was higher by 90% was obtained with 80% ethanol as the extraction solvent, compared to the use of 30% ethanol.

Similarly, Wang et al. [5] used 80% ethanol for CA extraction from cinnamon leaf powder followed by sonication at 60 °C for 2 h. A total of 15 components were separated within 14 min using UPLC-MS/MS with a C18 100 Å LC column (1.6 μm particle size, 100 × 2.1 mm ID) and a gradient mobile phase of acetic acid in water (A) and 0.025% acetic acid in methanol (B) at a ratio of 40:60 in the beginning, as well as a column temperature of 30 °C, flow rate of 0.3 mL/min and detection in MRM mode. Similar to a report by Huang and Chen [4], the main components found here to be present in cinnamon leaf extract included benzoic acid, quercetin, cinnamyl alcohol, CA, kaempferol-3-β-D-glucopyranoside, trans-cinnamic acid and eugenol, with the contents being 56.4, 16.6, 76.8, 17,985.2, 16.6, 387.4, and 183.4 μg/g, respectively.

In another study, Gutierrez et al. [59] employed UAE with the hydroalcoholic extraction of CA from *C. Zeylanicum* powder for subsequent analysis by liquid chromatography–tandem mass spectrometry (LC-MS/MS), using a C18 column (2.1 mm × 100 mm ID, 3.5 µm), a gradient mobile phase of water–formic acid (0.1%) (A) and a mobile phase of water–acetonitrile (B) to separate and identify 10 compounds, with CA and cinnamic acid contributing significantly to the total polyphenol content (310 mg gallic acid equivalent (GAE)/100 g).

#### 3.2.3. GC Method

Another method frequently used in cinnamon analysis is GC, which can be coupled with a flame ionization detector (FID) or MS for detection. Most GC techniques employ fused silica capillary columns with nitrogen [60], helium [52,64,65,66], or hydrogen [67] as the carrier gas. For example, Emami et al. [60] determined the CA in cinnamon bark using a 5MS-HP column (30 m long, 0.25 mm ID, 0.25 μm film thickness), with nitrogen as the carrier gas and the column temperature programmed to rise from 45 °C to 250 °C at 5 °C/min, CA was present at a high level, accounting for 33.74%. Yitbarek et al. [62] evaluated optimal extraction conditions for CA from *C. verum* leaf by steam distillation followed by GC with a capillary column (HP-5MS UI, 30 m long, 0.25 mm ID, 0.25 μm film thickness) and helium as the carrier gas, as well as a column temperature programmed to rise from 120 °C to 260 °C at a rate of 10 °C/min. The main bioactive compounds of cinnamon leaf essential oil extracted at optimal conditions were identified to be eugenol and CA, accounting for 60.68% and 33.94%, respectively.

More recently, Fazillah et al. [61] developed a gas chromatography–mass spectrometry (GC-MS) method for the determination of CA in cinnamon bark (*C. burmannii*). Initially, samples were subjected to microwave-assisted extraction by various solvents including distilled water, ethanol and distilled water-n-hexane (1:1), with a sample to solvent ratio of 1:6 and power level of 300 watts for 30 min. A high yield of cinnamon (5.6%) was obtained with ethanol as the extraction solvent. Yu et al. [52] analyzed bioactive components of hydrosol, a product from *C. cassia*, by steam distillation, and reported that CA was the most abundant one, accounting for 82.1% with the sample to water ratio at 1:10 (g/mL) for extraction and subsequent analysis by GC-MS using an HP-5MS column (60 m long, 0.25 mm ID, 0.25 μm film thickness), with helium as the carrier gas, a flow rate of 1 mL/min, an injection volume of 1 μL, an MS temperature of 180 °C, and a column temperature programmed to rise from 50 °C to 180 °C at 2 °C/min and then to 300 °C at 20 °C/min.

For the analysis of CA in three cinnamon species including *C. verum*, *C. cassia* and *C. loureiroi*, Rehman et al. [63] developed a GC-MS (splitless mode) method using a HP-5MS column (30 m long, 0.25 mm ID, 0.25 μm film thickness), with helium as the carrier gas, a flow rate of 1.2 mL/min and an injection volume of 1 µL for comparison with GC using an HP-5 19091J-413 capillary column (30 m long, 0.32 mm ID, 0.25 μm film thickness) coupled with an FID detector. Both GC-MS and GC-FID methods showed that oil samples extracted from different cinnamon species contained the highest level of CA (>94%), followed by cinnamyl acetate and 2-methoxycinnamaldehyde. This outcome has further revealed that both GC-MS and GC-FID are reliable methods for use in analyzing the contents of CA and its derivatives in essential oils extracted from cinnamon.

Taken together, most analytical methods of CA do not report the greenness profile, a term for evaluating if the analytical procedures employed are more environmentally friendly and safer to humans thanks to reductions in the amount and toxicity of solvents, waste, energy requirement and procedure steps, as well as miniaturization and automation [68,69]. As most reported methods are time-consuming, laborious, expensive and non-eco-friendly, there is an urgent need to replace the traditional extraction methods with a more advanced technique to enhance CA recovery from cinnamon. Critical evaluations of column type and length, film thickness, stationary phase as well as the column temperature condition are crucial for improving CA resolution and decreasing the analysis time required by GC techniques. Similarly, developing a sensitive, rapid, and sustainable HPLC method is necessary, and can be achieved through the selection of an appropriate column, mobile phase and detector.

## 4. In Vitro Release, Absorption, Pharmacokinetics, Bioavailability and Metabolism of CA

**Figure 3:**
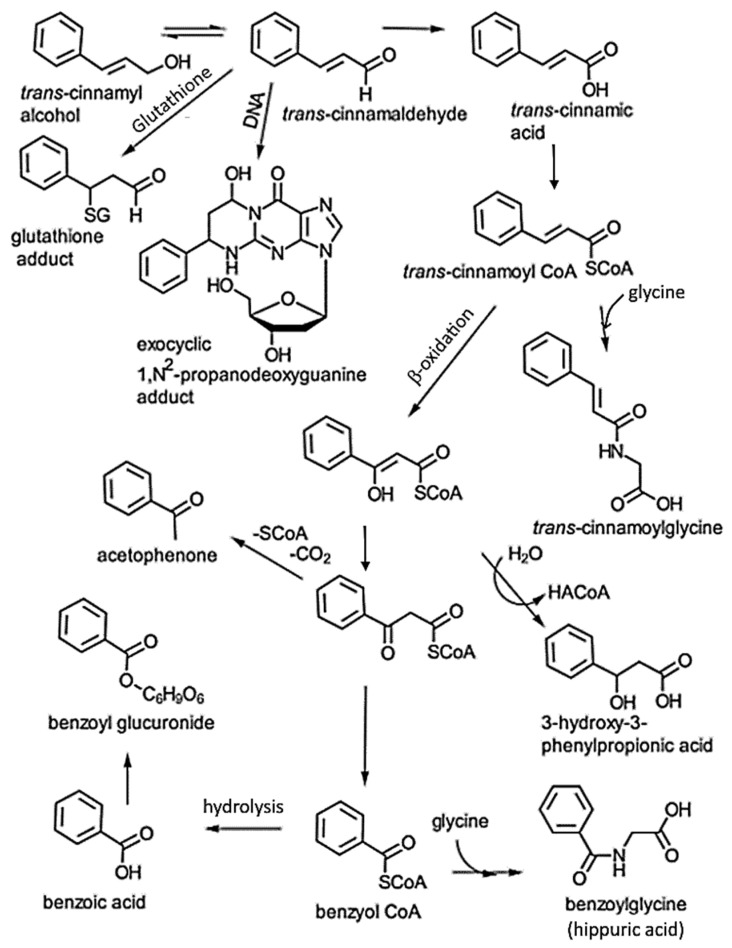
Metabolism of cinnamaldehyde (adapted with permission from Belsito et al. [77]).

The release of drugs or functional compounds under gastrointestinal conditions, intestinal absorption through endothelial cells, and entrance into systemic blood circulation are the key factors that determine the bioavailability and bioactivity of any therapeutic compound. In vitro release kinetics are usually performed under simulated gastric (acidic pH) and intestinal (pH 6.8 or 7.4) conditions, and the release of a drug or functional compound through a dialysis membrane at different time intervals can be determined by a suitable analytical method. For absorption, the Caco-2 cell is often used as an in vitro model, while the pharmacokinetic parameters such as T_max_ (time to attain maximum concentration in plasma), t_1/2_ (time to attain half the maximum concentration), C_max_ (maximum concentration in plasma) and AUC_0–∞_ (area under the concentration-time curve) for the subsequent determination of bioavailability using animal models can be carried out by collecting blood samples at different time intervals for the analysis of drugs or functional compounds in plasma. Several recent studies compared the in vitro release of CA from micro/nano-encapsulated CA and CA–polymer composites, demonstrating a sustained and higher rate of release of CA from the former possessing enteric properties, that is, low release under gastric conditions (acidic pH) and high release under the intestinal condition (pH 6.8 or 7.4) [19,20,70,71,72,73,74]. In addition, microemulsion, microcapsules, pellets and nanoemulsions prepared with CA were shown to exhibit a controlled CA release property under intestinal conditions, with the T_max_, C_max_, AUC_0–∞_ and relative bioavailability for micro/nano-encapsulated CA being higher than that for nonencapsulated CA [19,20,73,74,75]. CA undergoes a large number of metabolic transformations in vivo without long-term accumulation in rat tissues [76], with cinnamic acid being reported as the major intermediate metabolite of CA and cinnamyl alcohol (Figure 3). Cinnamic acid can then undergo esterification with coenzyme A to form cinnamoyl CoA ester, which in turn either conjugates with glycine or undergoes β-oxidation, resulting in the formation of benzoyl CoA for the subsequent generation of hippuric acid and benzoic acid, respectively, through conjugation with glycine and hydrolysis [13,77], implying that the β-oxidation pathway is the major pathway involved in the metabolic transformation of CA in animals (Figure 3) [77].

## 5. Antioxidant and Anti-Inflammatory Activities

### 5.1. Antioxidant Activity

Antioxidants are compounds capable of trapping and deactivating oxygen-centered radicals, and thus have been proven to be protective against various diseases, as free radicals are responsible for the oxidative damage of lipids, proteins and nucleic acids. However, many synthetic antioxidant compounds exert toxic and/or mutagenic effects on human health [78]. Consequently, naturally occurring antioxidants such as flavonoids from fruits, vegetables and leaves have been consumed for protection against diseases, including cancer and cardiovascular diseases, owing to their potential use in inhibiting free radicals and lipid peroxidation processes [78]. In several studies, cinnamon extract/oil and cinnamaldehyde isolated from cinnamon species have been shown to be effective in inhibiting reactive oxygen species (ROS) production through thioredoxin signaling pathways and the activation of the oxidative stress defense system, such as nuclear factor erythroid 2–related factor 2 (Nrf2) [79,80]. For instance, Choi et al. [81] showed that TCA could attenuate the apoptosis of mouse myoblast cells (C2C12) caused by mitochondria dysfunction and oxidative stress through suppressing the loss of mitochondrial membrane potential and the cytosolic release of cytochrome-C, as well as reducing the activity of caspase-3 and increasing the Bcl-2/Bax (B-cell lymphoma 2/Bcl-2-associated X protein) expression ratio in H_2_O_2_-stimulated cells.

One major drawback of cinnamon antioxidants is that they can be susceptible to oxidation. However, through the conjugation of CA with β-cyclodextrin, an enhanced antioxidant activity was shown for scavenging DPPH (2,2-diphenyl-1-picrylhydrazyl) and ABTS (2,2-azino-bis-3-ethylbenzothiazoline-6-sulphonic acid) radicals compared to β-cyclodextrin alone, with a rise in molar ratio of β-cyclodextrin-CA from 1:0 to 1:1 showing a greater effect due to enhanced CA solubility and antioxidant activity [82]. In another study, Yan et al. [21] prepared a TCA bioactive film through the conjugation of chitosan with polyvinyl alcohol and TCA, resulting in a strong antioxidant activity, as evident by the elevation of ferric reducing power and the DPPH radical scavenging activity at its highest CA level. More specifically, following a rise in CA concentration from 0.5% to 1.5%, the DPPH radical scavenging activity increased from 19.22% to 31.43%, which is 3-fold higher than that observed for pure chitosan. This observed antioxidant activity of the bioactive film can be associated with the CA aromatic structures, as well as the hydroxyl groups and benzene rings of essential oils. Similarly, Dong et al. [83] developed a chitosan/alginate dialdehyde trilayer film containing 10% CA nanoemulsion, and observed a higher DPPH scavenging activity (>80%) compared to that (21%) without CA nanoemulsion. Additionally, the antioxidant activity showed a dose-dependent increase with the increase in CA dose. These findings reveal that cinnamon oil can be incorporated into nanoparticles to facilitate its application in food packaging as an antioxidant.

Additionally, cinnamon extract, cinnamon oil and CA encapsulated into metal and polymer-based nanoparticles were demonstrated to exhibit high antioxidant activity due to the increased water solubility, large surface-to-volume ratio and possible synergistic effects. For instance, in a study dealing with the antioxidant activity of silver nanoparticles (AgNPs) synthesized using *C. zeylanicum* bark extract in different polar solvents, a remarkable impact on the antioxidant activity of the extract and the formation of nanoparticles with different shapes and sizes were shown [84]. The antioxidant activity followed the order ethanol > water > DMSO. A high level of CA-loaded nanoparticles may be responsible for the high antioxidant activity. In a later study, El-Baz et al. [85] synthesized Ag nanoparticles with cinnamon extract with a mean particle size of 6–35 nm and zeta potential of −12.3 mV, and they possessed a higher antioxidant activity than all the other cinnamon samples, while a significant rise in antioxidant enzymes’ activity (superoxide dismutase (SOD), glutathione (GSH), catalase (CAT), glutathione peroxidase (GPx)) was observed in samples treated with nanoparticles. More recently, Lopez-Cano et al. [86] synthesized different types of nanoparticles (CaCO_3_, TiO_2_, and Al_2_O_3_) and, compared with their pure form loaded with cinnamon oil, it was shown Al_2_O_3_ modified with cinnamon essential oil (CEO) exhibited a high degree of inhibition against DPPH radicals of 55%, followed by CaCO_3_-CEO (35%) and TiO_2_-CEO (28%). Also, Al_2_O_3_-CEO enhanced the thermal and mechanical properties of polylactic acid films, which can be employed in the active packaging of products to increase their antioxidant performance.

Furthermore, the antioxidant activities of nanoparticles loaded with cinnamon oil were shown to be higher than those of free cinnamon oil, as demonstrated by Su et al. [87], who synthesized chitosan nanoparticles from cinnamon oil with a mean particle size of 190–340 nm, encapsulation efficiency of 4.6–32.9%, and loading capacity of 0.9–10.4%, via an oil-in-water emulsification and ionic gelation method. It was shown that the encapsulation of cinnamon oil into chitosan nanoparticles with an encapsulation efficiency of 32.9% could elevate the scavenging of DPPH and hydroxyl radicals by 56.9% and 53.4%, respectively, which can be attributed to the synergistic effect of chitosan and the cinnamon oil nanoparticle. Cartaya et al. [88] synthesized CA in a nanoformulation based on the micellization behavior of the pluronic polymer L101 in solution; a mean particle size ranging from 100 to 200 nm with an entrapment efficiency of 7.6% was shown. Moreover, the treatment of vascular smooth muscle cells with CA only significantly increased SOD activity, while under the treatment with the CA nanoformulation, the total glutathione (GSH) and SOD activities were significantly raised. Thus, CA may be incorporated into micelles for possible delivery to target sites, enabling the treatment of vascular injury.

### 5.2. Anti-Inflammatory Activity

Inflammation plays a pivotal role in the host’s immune response to harmful stimuli such as pathogens, irritants and damaged cells, helping to mitigate the primary cause of infection or tissue injury, as well as eliminating apoptotic/necrotic cells and damaged tissue for the initiation of tissue repair [89]. Immune cells, including macrophages, neutrophils, and lymphocytes, respond to infectious agents by modulating an inflammatory response via the NF-κB pathway for producing various inflammatory mediators [89]. These mediators can cause vascular endothelial cells to attract circulating blood cells for inducing hypothalamic neurons to reset the body temperature and launch the glucocorticoid response through the hypothalamic–pituitary–adrenal axis [90]. Consequently, an impairment in the secretion of inflammatory mediators by immune cells can lead to a variety of chronic inflammatory disorders. On the contrary, an excessive host inflammatory response can damage the nerves and tissues, causing severe pain [91]. Commonly used anti-inflammatory drugs possess both limited efficiency and increased side effects. Thus, bioactive compounds possessing the capacity to regulate the inflammatory response of macrophages can be regarded as potential alternative therapeutic agents for exerting anti-inflammatory effects.

In a human macrophage model stimulated with *Aggregatibacter actinomycetemcomitans*/*E. coli* lipopolysaccharide (LPS), the cinnamon fraction was found to dose-dependently enhance anti-inflammatory functions [89]. In the U937-3xκB-LUC cell line, the cinnamon fraction was also shown to prevent LPS-induced NF-κB activation by lowering luciferase activity, making it possible to control NF-κB activation. In addition, the cinnamon fraction reduces LPS binding to monocytes, which may contribute to its anti-inflammatory properties. In a later study, Pagliari et al. [92] evaluated the anti-inflammatory effect of cinnamon extract after in vitro digestion through a Caco-2 cell model. The exposure of Caco-2 cells to TNFα/IL-1β induced a rise in phosphorylated NF-ĸB by 3-fold in comparison with untreated Caco-2 cells. Following inflammation, a reduction of 30% in p65-NF-ĸB phosphorylation and 25% in IL-8 was found in the group pre-treated with 46 µg/mL of cinnamon-digested extract, revealing the anti-inflammatory function of cinnamon. In addition to NF-ĸB, one of the most studied key markers of inflammation is COX-2, which catalyzes arachidonic acid conversion in the inflammatory lipid mediator’s prostaglandins (PGE2). Interestingly, the group pre-treated with 46 µg/mL of cinnamon-digested extract reduced COX-2 by 35% under a pro-inflammatory condition.

In an in vivo experiment carried out by Abeysekera et al. [22], a marked reduction in the mRNA expression of inflammatory cytokines (cyclooxygenase-1 (COX-1) and cyclooxygenase (COX-2)), nitric oxide radicals and superoxide radicals related to stress signaling pathways was observed for both ethanolic and dichloromethane–methanol leaf extracts of cinnamon. Moreover, dichloromethane–methanol leaf extract showed the highest inhibition of COX-1 at 6.62 ± 0.85 µg/mL and COX-2 at 44.91 ± 3.06 µg/mL compared to ethanolic cinnamon leaf extract. In another study, CA applied at doses of 20 and 50 µmol/L was shown to inhibit the expression of IL-1β, IL-6 and TNF-α and the activation of the toll-like receptor 4/myeloid differentiation primary response 88 (TLR4/MyD88) signaling pathway in LPS-induced osteoarthritis fibroblast-like synoviocytes [93]. Esmaeili et al. [91] prepared cinnamon and clove essential oil nanoemulsions with mean particle sizes of 28 ± 6 nm and 12 ± 3 nm, respectively, and evaluated their anti-inflammatory effects in rats using a paw edema test. The cinnamon nanoemulsion was more effective than clove essential oil nanoemulsion against the carrageenan-induced inflammation of paw edema, especially at the 4th and 5th hour of the test, and may be used as an anti-inflammatory agent or in promising therapeutics for relieving the symptoms of pain and inflammation. Apparently, the higher anti-inflammation effect of cinnamon nanoemulsion can be attributed to the presence of a high level of CA, which can penetrate into paw tissues more readily due to the presence of a small mean nanoparticle size (28 ± 6 nm).

## 6. Anticancer Activity

Apoptosis, a phenomenon of programmed cell death, can remove unwanted cells. Apoptosis occurs via four pathways, as follows: the intrinsic pathway through mitochondria, the extrinsic pathway through death receptors, the endoplasmic reticulum stress-mediated pathway and the granzyme B-mediated pathway. The anti-cancer activities of *C. zeylanicum* and CA were studied via different in vitro tests on oral cancer cells SCC-4, SCC-9, and SCC-25 [94]. In SCC-9 cells, the cinnamon extract treatment (200 μg/mL) induced early apoptosis from 5% to 41.1%, and late apoptosis from 6.3% to 22%, while CA induced early apoptosis from 5% to 90.5% and late apoptosis from 6.2% to 39.7%. Reduced expressions of COX-2, vascular endothelial growth factor (VEGF), NF-κB, and Bcl-2 were observed for both extract and CA treatments. In addition, both the cinnamon extract and CA treatments could prevent the cytoplasmic translocation and invasion of NF-κB on these cell lines, with the downregulation of Bcl-2 and NF-κB being possible factors for the induction of apoptosis in oral cancer cells [94]. Table 2 shows the anticancer effects of cinnamon extract and CA on various cancer cells and tumors in animal model. 

Many studies have demonstrated that in the presence of CA, the apoptosis of tumor cells can occur through the activation of different pathways. CA at different levels (2.5, 5, 10, 20, and 40 μg/mL) was shown to inhibit the proliferation of MDA-MB-231 breast cancer cells, with the half maximum inhibitory concentration (IC_50_) at 16.9 μg/mL after 24 h treatment and 12.23 μg/mL after 2-day treatment, and the apoptosis percentages were 9.5%, 10.5%, and 22.5% with CA doses at 10, 15, and 20 μg/mL, respectively [95]. As a result, the findings of this study demonstrate that a total of 83 cell biological processes and 37 pathways can be associated with breast cancer, with the phosphoinositide 3-kinase (PI3K-Akt) and peroxisome proliferator-activated receptor pathways being closely related to breast cancer cell apoptosis [95]. After treating prostate cancer-related fibroblast cells with CA at 150 μM for 17 h, Han et al. [96] found that CA has the ability to trigger endogenous apoptosis by inhibiting the function of mitochondrial glutathione. The reduction of the intracellular antioxidant GSH is another key that may cause cancer-related fibroblast apoptosis induced by CA, as cancer cells were not readily eliminated due to the protection of tumor cells from attack by ROS in the presence of GSH [96]. Aminzadeh et al. [97] reported that following the treatment of bladder cancer cells (5637) with CA for different time lengths, the apoptosis occurred in a dose-dependent manner through the suppression of the expression of epidermal growth factor receptor 2, heat shock protein transcription factor-1, and the lactate dehydrogenase A pathway.

Several studies have also demonstrated the anticancer activity of CA in combination with a chemotherapeutic drug. The treatment of CA or doxorubicin (DOX) alone was shown to inhibit glioblastoma cells (U87MG), with the IC_50_ of doxorubicin and CA being 5 and 11.6 µg/mL, respectively, based on MTT assay [98]. However, combinations of CA and doxorubicin at doses of 8 μg/mL each were shown to exhibit much higher inhibition efficiencies towards U87MG cells with an IC_50_ value of 1.75 µg/mL. Furthermore, a rise in sub-G1 ratio, caspase-3 and caspase-9 activities, and Bax expression, and a decline in mitochondrial membrane potential, were shown following CA treatment, with the apoptotic effect being reinforced when co-administered with doxorubicin, apparently due to a synergistic effect. It is worth pointing out that the decrease in mitochondrial membrane potential can be caused by a knockdown in lactate dehydrogenase, resulting in the elevation of ROS and an increase in the release of cytochrome C, which ultimately leads to apoptosis. Thus, it is possible to treat patients with glioblastoma tumors with a combination of CA and doxorubicin. However, the side effects need to be further evaluated. Park et al. [99] found that a combination treatment of CA (200 µM) with hyperthermia (43 °C) induced apoptosis in lung cancer cells (A549) through the regulation of the mitogen-activated protein kinase (MAPK) family and the elevation of ROS. Additionally, this combination treatment decreased expressions of vascular endothelial growth factor, cyclin D1, matrix metallopeptidase (MMP)-9 and MMP-2.

For a novel cancer therapy, the selective activation of stimuli-responsive polymers in the tumor microenvironment has been recently explored. However, most of them show inadequate responses due to insufficient endogenous triggering agents. Consequently, a ROS-responsive CA-polythioacetate conjugate with self-amplified chain-shattering polymer degradation was developed by Zong et al. [112] for promoting chemoimmunotherapy and inhibiting cancer cell growth. A CA/polythioacetate conjugate in the presence of endogenous ROS can undergo cleavage, releasing CA, which in turn generates more ROS via mitochondrial dysfunction for the subsequent exponential degradation of polythioacetate. Also, the incorporation of doxorubicin with CA/polythioacetate can further amplify oxidative stress due to ROS production, facilitating immunogenic tumor cell death during chemoimmunotherapy. Likewise, Tu et al. [100] synthesized a ROS-self-amplifying degradable CA using a polythioacetal polymer with an average particle size of 96.2 nm and zeta potential of −10.4 mV, in which the thioacetal in polythioacetal polymer can act as an ROS-responsive group, while CA acts as the ROS generating agent. CA was released for the production of more ROS via mitochondrial dysfunction in colon cancer cells CT26, leading to amplified polymer decomposition. Only modest tumor inhibition was shown in the CA group, while CA conjugated with NPs revealed a much higher inhibition (83.9%).

A kind of controlled cell death measured by the accumulation of lipid peroxides to fatal levels is known as ferroptosis. Most cancer cells are highly vulnerable to ferroptosis as a large amount of ROS can be generated. For this reason, cancer cells rely on GSH to decrease lipid peroxide formation via glutathione peroxidase 4 (GSH peroxidase 4) for survival. Yan et al. [101] prepared RAS-selective lethal small-molecule 3@acid-responsive nanoparticles (RSL3@PCA) to simultaneously decrease the intracellular GSH of breast cancer cells 4T1 and prevent the activity of GPX 4 (GSH peroxidase 4), thereby enhancing cancer cell ferroptosis. RSL3@PCA was synthesized by loading a selective inhibitor of GPX 4 into RSL3@PCA. This nanomaterial gradually degraded to deliver CA and the incorporated RSL3 for the subsequent production of lipid peroxides in cells, leading to ferroptosis. By using a 4T1 cell xenotransplantation model and cytotoxicity method, RSL3@PCA was shown to induce a better inhibition of cancer cells without toxicity to normal organs/tissues.

**Figure 4:**
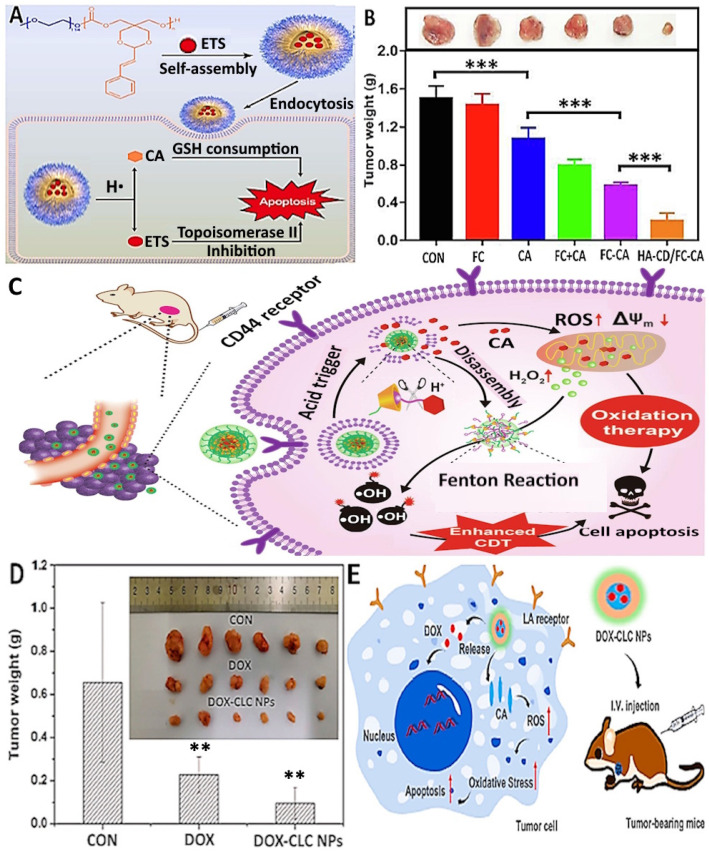
Anticancer mechanism of different polymer-encapsulated and drug-functionalized forms of CA in different animal models. (**A**) Synthesis and apoptosis mechanism of self-assembled etoposide (ETS)-encapsulated biocompatible acid-responsive polyethylene glycol-polycarbonate CA (PEG-PCA), with the hydrolysis of acetal linkage increasing the hydrophilicity and degradation rate of the polycarbonate backbone, facilitating the release of ETS and CA into the cytosol of target cancer cells and exerting a synergistic effect on alleviating the deficiency of ETS by consuming GSH. (**B**,**C**) Inhibition of tumor growth by ferrocene (FC)-modified CA encapsulated into β-cyclodextrin-functionalized hyaluronic acid (HA-CD/FC-CA) against breast cancer cell 4T1 tumor-bearing mice, along with (**B**) the in vivo therapeutic mechanism of pH/redox dual-responsive supramolecular HA-CD/FC-CA for synergistic chemo/chemodynamic therapy via amplified oxidative stress, decreased mitochondrial membrane potential and cascaded Fenton reaction (**C**). (**D**,**E**) Inhibition of tumor growth by doxorubicin (DOX) and a hybrid nanoparticle of chitosan-modified CA and chitosan-modified lactobionic acid in combination with DOX (DOX-CLC NPs) towards liver cancer cell H22 tumor-bearing mice, along with (**D**) the synergistic inhibition mechanism exerted by DOX-CLC NPs (**E**). *** in (**B**) represents statistically significant data at *p* < 0.001. ** in (**D**) represent statistically significant data for DOX and DOX-CLC NPs treatment compared to control at *p* < 0.01. CA, cinnamaldehyde; CON, control; GSH, glutathione; ROS, reactive oxygen species; I.V., intravenous; CD44, cell surface receptor; CDT, chemodynamic therapy; ΔΨm, mitochondrial membrane potential. Adapted with permission from Wu et al. [103], Xu et al. [104], and Zhou et al. [108].

Moreover, Zhu et al. [102] developed glutathione (GSH)-responsive triphenylphosphine (TPP) and hyaluronic acid (HA)-conjugated CA-mesoporous organosilica nanoparticles (MON-CA-TPP@HA), which was reported to be a more effective therapeutic approach for cancer treatment via inducing immunogenic cell death and the apoptosis of breast cancer cells 4T1. An in vitro experiment indicated that this nanodrug can actively target cancer cells with the help of hyaluronic acid, enabling subsequent penetration into cancer cells for precise linkage with mitochondria via triphenylphosphine residues. Upon cleaving the disulfide linkage in NPs triggered by the over-expressed glutathione within tumors, CA can be released to induce the production of ROS in situ, surrounding the mitochondria to thus enhance oxidative stress for apoptosis promotion and the immunogenic cell death of breast cancer cells. More recently, Wu et al. [103] developed biocompatible acid-responsive polycarbonate (PEG-PCA) containing CA and self-assembled to form nanoparticles that encapsulated ETS (etoposide), a potent GSH-depleting agent, using a female BALB/c mice abdomen model. The results showed that ETS@PCA could respond to the acidic tumor microenvironment by releasing CA to deplete GSH and releasing ETS to inhibit topoisomerase II, an enzyme that is essential to the survival of proliferating cells through DNA regulation by under- and overwinding, thereby exhibiting superior cytotoxicity compared to free ETS (Figure 4A).

Hybrid polymer conjugates of CA and their micro/nanoparticles were also evaluated for their anticancer efficiency against several cancer cell types. For instance, Xu et al. [104] developed a prodrug using ferrocene-modified CA for encapsulation into β-cyclodextrin-functionalized hyaluronic acid, which was active against breast cancer cells (4T1 and MCF-7) at different concentrations (2.5, 5, 10, 25, 50, 100 and 250 μM), with an average particle size of 110 nm and zeta potential of −19.5 mV. The total apoptotic rate of the NPs group rose to 45.6%, which can be attributed to the elevated level of intracellular H_2_O_2_, revealing the efficiency of CA in causing oxidative damage to cancer cells. Also, the combination of oxidative stress enhancement and the generation of more hydroxyl radicals via the Fenton reaction can improve the anti-tumor effect in BALB/C mice [104]. Figure 4B,C illustrates the inhibition of tumor growth in breast cancer cell 4T1 tumor-bearing mice by use of ferrocene (FC)-modified CA encapsulated in β-cyclodextrin-functionalized hyaluronic acid (HA-CD/FC-CA) along with the therapeutic mechanism of pH/redox dual-responsive supramolecular HA-CD/FC-CA for synergistic chemo/chemodynamic therapy via amplified oxidative stress, decreased mitochondrial membrane potential and a cascaded Fenton reaction.

One strategy to fully explore the efficacy of CA in cancer treatment is to find an agent that can be used in combination with CA to decrease cellular GSH while enhancing ROS production. Liu et al. [105] prepared a GSH-depleting agent (diallyl trisulfide) encapsulated into a poly(lactic-co-glycolic) acid–polyethylene glycol copolymer with CA to enhance cancer treatment efficiency, with the mean particle size being 145 nm and the zeta potential being 21.5 mV. With CA at 100 mM and diallyl trisulfide at 50 mM, it was shown to possess the best synergistic effect in inhibiting the growth of MCF-7 cells, as evident from the low viability (12.3%) when compared to individual CA and diallyl trisulfide, with high viability values of 83.7% and 81.5%, respectively. Furthermore, ROS production was raised to a much higher level following the combination treatment.

Barrera-Martinez et al. [106] developed chitosan microparticles (CMPs) loaded and unloaded with TCA and a crosslinking agent (sodium tripolyphosphate), with the mean particle size and zeta potential being 117–478.5 nm and 27.8–103.5 mV, respectively, while the entrapment efficiency of TCA in CMPs was 9.1%. Following treatment with TCA-loaded CMPs (0–50 µM) for 24 h, a cytotoxicity of 64.5% and 67.6% was shown for HeLa and MDCK cells, respectively, but this was raised to 77.9% and 81.1% after 48 h of treatment. Obviously, the chitosan in CMPs can interact with the tumor microenvironment to inhibit cell growth or induce apoptosis, while CA can be a potential candidate for inhibiting cancer cell growth through the elevation of intracellular ROS levels for different types of human cancer cells, including breast cancer cells MCF-7 and MDA-MB-231 and lung cancer cells A549 [113]. Similarly, Purushothaman et al. [107] synthesized a casein–calcium ferrite hybrid biopolymeric carrier conjugated with biotin for the delivery of CA for the treatment of lung cancer (A549 cells), with the average particle size varying from 117 to 478 nm, and the zeta potential ranging from +27.8 to +103.5 mV. The in vitro cell viability results show that this carrier significantly increased the anticancer activity, with IC_50_ reduced from 45 μg/mL for CA alone to 2 μg/mL for this carrier.

In a later study, Zhou et al. [108] developed a hybrid (CLC NPs) from lactobionic acid-modified chitosan and CA-modified chitosan, both of which possessed ROS scavenging and tumor-targeting abilities. Lactobionic acid was shown to enhance the cellular accumulation and tumor-targeting capacity of these NPs, while CA promoted cell apoptosis via mitochondrial permeability transition, ROS production, and caspase activation. Both in vitro and in vivo studies have indicated that CA-based nanoparticles showed a significant synergistic anticancer effect, possessing a great potential to be developed as antitumor drug carriers. Furthermore, NPs showed the most remarkable ability in inhibiting the growth of human liver cancer cells HepG2 using multicellular spheroids for the cell culture, with their average diameter decreasing to 128 μm on day 7. Three-dimensional cell cultures with spheroid formation are amongst the best described models for 3D cell cultures due to their simplicity and similarity to real physiological tissues, as spheroids are self-assembled agglomerates of cell colonies that naturally resemble avascular environments, with gradients of oxygen, carbon dioxide, nutrients, and water-soluble wastes [114]. As mentioned above, CLC NPs improved both the uptake and enrichment of NPs in cells by targeting the functions of lactobionic acid and CA release, producing a synergistic anti-cancer effect. Moreover, an in vivo study demonstrated the most significant decrease in tumor weight for an H22 tumor-bearing mice group intravenously injected with DOX-CLC NPs, followed by a free DOX group, compared to the control (Figure 4D,E) [108].

In contrast to CA-encapsulated polymers, CA-conjugated polymers can not only control CA delivery, but also act as drug release systems. For instance, CA can be released from CA-conjugated polymers as an oxidative stress-inducing chemotherapeutic agent to enhance anticancer activity. CA-conjugated polymeric micelles were also developed as prodrugs loaded with an anticancer agent. The resulting complex was shown to self-assemble for the formation of polymeric micelles to deliver CA for the induction of human colon cancer cell SW620 apoptosis, but at low pH, micelle breakdown occurred, caused by the cleavage of imine bonds. In comparison with normal cells, the higher cytotoxicity of CA-conjugated prodrug micelles towards tumor cells could be due to the elevated oxidative stress suffered by tumor cells [109].

In recent years, various nano-delivery systems such as nanoparticles, nanogels, nano liposomes, nanocluster and micelles have been developed for tumor treatment. The advantages of nano-formulations include prolonging circulation in vivo, solubilizing hydrophobic agents, reducing systemic toxicity, and having a targeting site of the tumor tissue for accumulation. However, only a few of them have been approved by the USFDA. The main limitations of these novel systems are the complexity of the human body and the heterogeneity of cancer cells. One of the anti-metabolic drugs used in clinical tumor treatment is 5-fluorouracil (FU). Fang et al. [23] developed a carrier-free nano-drug composed of an FU and CA conjugated prodrug, with a mean particle size of 17.5 nm and PDI of 0.121. In a male ICR mice model, the self-assembled FU–CA nanoparticles (NPs) had higher tumor growth inhibition than a mixture of FU and CA, with a lower systemic toxicity being shown following a decline in dose for both. The total apoptosis ratio was 80.7% for FU–CA NPs with a dose at 385 µM. Thus, FU–CA NPs showed the highest anti-cancer activity, which can be attributed to their synergistic effect. Chang et al. [110] developed new anticancer NPs with a mean particle size of 172 nm by preparing a derivative of CA with anthraniloyl hydrazine, followed by conjugating with bovine serum albumin (BSA). The intracellular uptake of BSA–CA NPs was shown to inhibit lung cancer cells (A549) by >50%, malignant melanoma cells (A375) by >50% and human hepatoma (HepG2) by >50%, as well as human laryngeal squamous cells (Hep2) by >80%. In a later study, Asadi-Yousefabad et al. [111] synthesized gelled-oil NPs by loading CA and/or tannic acid into the oil and aqueous phases, respectively, with mean particle sizes from 77 to 163.8 nm and zeta potentials from −33 to −38 mV, while the encapsulation efficiencies of CA in CA gelled-oil NPs and CA-tannic acid-gelled-oil NPs were 94.25% and 97.76%, respectively. The EC_50_ values of CA gelled-oil NPs, tannic acid gelled-oil NPs and CA-tannic acid gelled-oil NPs for scavenging DPPH radicals were 223.11, 182.51 and 107.06 μg/mL, respectively, while CA-tannic acid gelled-oil NPs were shown to be the most effective in inhibiting breast cancer cell MCF7 and lung cancer cell A549, at 81.66% and 57.54%, respectively.

Moreover, a combination of CA and chemotherapeutics was found to raise the level of ROS in tumor cells, reinforce oxidative stress, and further generate a notable synergistic effect with chemotherapeutics. The elevation of the oxidative stress of tumor cells caused by ROS generation is a practical mechanism for inducing the apoptosis of cancer cells. The incorporation of CA into iron oxide magnetic nanoparticles that were functionalized with folic acid (CA-IONPs/FA) for active drug delivery was studied in breast cancer cells E0771-induced mouse medullary breast adenocarcinoma (0–1.25 μg/mL) by Shetty et al. [24]. The average nanoparticle size was 10 nm, with a 20% loading of CA into NPs based on thermogravimetric analysis. FA incorporation was shown to enhance the uptake of NPs in tumor cells, with localization in both the nucleus and the cytoplasm. Interestingly, tumor-bearing mice treated with CA-IONPs/FA and free CA showed tumor volumes that were reduced by 3.2- and 1.6-fold, respectively, as well as tumor weights reduced by 3.2- and 1.1-fold, compared to the control. The results of the in vitro study also show that CA-IONPs/FA induced the apoptosis of breast cancer cells (MCF7, MDAMB231) mainly through the elevation of mitochondrial depolarization, calcium ion release, and caspase expression through active internalization. It is worth pointing out that mitochondrial depolarization acutely promotes Ca^2+^ alternans via the redox effect of ROS (ROS elevation), and chronically via ATP reduction.

## 7. Alleviation of Metabolic Syndromes

Metabolic syndrome has become a critical public health problem, with a rising prevalence worldwide (10–84%), and it is associated with overweight, obesity, cardiovascular disease, type 2 diabetes (T2DM) and non-alcoholic fatty liver disease [115]. The factors contributing to metabolic syndrome include central adiposity, hypertension, increased levels of triglyceride, total cholesterol and low-density lipoprotein (LDL) cholesterol, and reduced levels of high-density lipoprotein (HDL) cholesterol, as well as dysglycemia, an unbalanced redox state, endothelial dysfunction, insulin resistance, a low-grade proinflammatory state and a hypercoagulable/prothrombotic condition [115,116]. Table 3 summarizes the effects of cinnamon extract, cinnamon oil and CA on various metabolic syndromes including obesity, cardiovascular disease and diabetes, using in vitro and in vivo models.

### 7.1. Anti-Obesity Activity

Obesity and overweight are the major epidemic diseases caused by imbalance between calorie intake and energy consumption, with 39% and 13% of the world’s population being affected, respectively [117,132]. As several commercial drugs possess limited therapeutic effects or exert severe side effects [27], several natural bioactive elements including CA were explored. The transient receptor potential channel A1 (TRPA1) agonist has been reported to have potential TRPA1 dependency in the context of its CA-induced effect on adipogenesis. Moreover, adipogenic transcription factors (PPARγ and members of the C/EBP family) have been recognized as key modulators in lipid accumulation and adipogenesis. Hoi et al. [117] investigated the molecular mechanisms regulating lipid metabolism and adipocytes 3T3-L1 by CA. 3T3-L1 cells are a popular model of fibroblast-like cells that can be differentiated into adipocytes and are used to perform studies on compounds that influence adipogenesis. Furthermore, 3T3-L1 cells of the adipocyte morphology can increase the synthesis and accumulation of triglycerides and acquire the singlet ring appearance of adipose cells. The triglyceride content, lipid accumulation and expression levels of adipogenic transcription factors including PPARγ, C/EBPα and C/EBPβ were analyzed in 3T3-L1 cells treated with CA and cinnamyl isobutyrate, with the latter being shown to decrease the phospholipid accumulation and triglyceride content by 20.7 ± 2.05% and 21.4 ± 2.56%, respectively, after 12-day treatment at 30 μM. But for CA at the same dose (30 μM), it reduced triglyceride content by 37.5 ± 1.81% and phospholipid accumulation by 28.7 ± 1.83%, indicating CA could be a more potent antiadipogenic compound than cinnamyl isobutyrate. Moreover, both cinnamyl isobutyrate and CA inhibited lipid accumulation through the downregulation of PPARγ, C/EBPα, and C/EBPβ, demonstrating their impacts on the modulation of adipogenic signaling cascades. A decreased triglyceride content also implied the involvement of TRPA1 in the antiadipogenic effect of CA. Conversely, no TRPA-1 involvement in the antiadipogenic effect of cinnamyl isobutyrate was shown, which may be due to the lower impact of cinnamyl isobutyrate on lipid accumulation when compared to CA.

To demonstrate this phenomenon, Neto et al. [118] explored the potential use of CA as a visceral adiposity therapeutic in a rat model of early obesity. The treatment of male Wistar rats (60-day age) with CA (40 mg/kg of body mass/day) manifested a reduction in serum triglyceride (71.81 ± 5.09 mg/dL), accompanied by a lower mRNA transcription of sterol regulatory element-binding transcription factor 1 (Srebf1) and acetyl-CoA carboxylase alpha (Acaca) in the liver. The decreased adiposity may be caused by fat oxidation within the visceral adipose tissue promoted by CA. Specifically, CA resulted in a high protein expression of the LC3II/I ratio and low sequestosome 1 (Sqstm1) mRNA expression, indicating a possible stimulatory impact of CA on autophagy. In addition, CA treatment leads to lower phospho-eIF2α (eukaryotic translation initiation factor 2) and IRE1α (inositol-requiring enzyme 1α) expression, indicating decreased endoplasmic reticulum stress, which may be associated with a reduction in insulin resistance.

As mentioned above, there are many metabolic syndromes, such as central adiposity, a high level of triglyceride, a low level of HDL cholesterol, and hypertension. Many studies have revealed that CA may have a beneficial role in the treatment of vascular disorders linked with metabolic syndromes. In a study dealing with Wistar rats fed with a high-fat plus sucrose diet (WHFDS) in the presence of CA at 20 mg/kg/day for 8 weeks, reductions in triglyceride, adiposity index, free fatty acid, and total cholesterol by 1.3-, 2.56-, 2.76-, and 1.33-fold, respectively, were shown compared to the WHFDS treatment without CA. Furthermore, CA was able to lower pro-inflammatory gene expression (CD11c, CD11b, F4/80 and TNF) and vascular oxidative stress in the WHFDS treatment, illustrating the optimization of endothelial cell function through the activation of the Nrf2 pathway [26]. In another study, high-fat diet (HFD)-induced atherosclerosis in rats was treated with CA at a dose of 20 mg/kg for 10 weeks [29], and the levels of serum total cholesterol, triglyceride, LDL, and free fatty acid were reduced substantially, accompanied by a decline in serum creatine kinase, creatine kinase-MB, lactate dehydrogenase, and aspartate aminotransferase, which play key roles in triglyceride synthesis. Thus, the treatment of HFD-fed rats with CA may be protective against obesity and cardiovascular disease.

**Figure 5:**
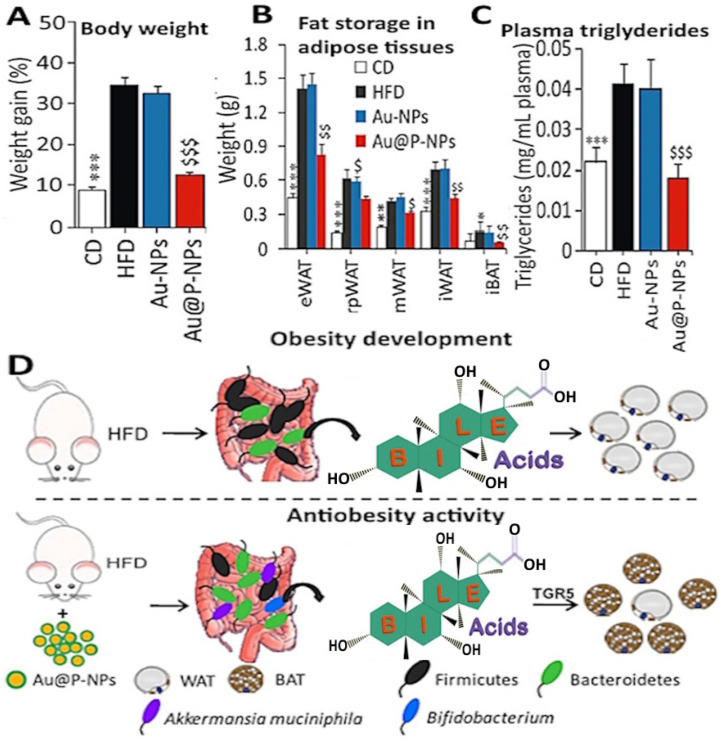
Anti-obesity effects of cinnamon extract and cinnamaldehyde on animal models. The effects of cinnamon extract-functionalized gold nanoparticles (Au@P-NPs) on body weight (**A**), fat accumulation in adipose tissue (**B**) and plasma triglycerides (**C**) of chow diet (CD) or high-fat diet (HFD)-induced obese mice, along with the anti-obesity mechanism of Au@P-NPs (**D**), illustrating that the administration of Au@P-NPs increased energy expenditure and altered bile acid profile via gut microbiota in HFD-induced obese mice, converting WATs into BATs. *, **, and *** represent statistically significant data for CD compared to HFD-fed mice at *p* < 0.05, *p* < 0.01 and *p* < 0.001 respectively. $, $$ and $$$ represent statistically significant data for Au@P-NPs treatment compared to HFD-fed mice at *p* < 0.05, *p* < 0.01 and *p* < 0.001 respectively. AuNPs, gold nanoparticles; Au@P-NPs, *C. verum* extract-functionalized gold nanoparticles; WATs, white adipose tissues; BATs, brown adipose tissues; eWAT, rpWAT, mWAT, iWAT, iBAT, indicators of fat accumulation in adipose tissue. Adapted with permission from Sharma et al. [27].

Sharma et al. [27] investigated the potential of *C. verum*-derived bioactive-functionalized gold NPs (Au@P-NPs) for use in anti-obesity therapeutics by using eight-week-old male Swiss mice given a dose of 10 mg/kg; a decrease in fat deposition, metabolic inflammation and endotoxaemia in HFD-fed mice was shown, accompanied by losses of body weight of 3.7%, 4.9% and 2.1% after the post-colonization of gut microbiota on days 1, 2 and 3, respectively, as well as a reduction in plasma triglyceride (Figure 5A–C). Furthermore, compared to the control (HFD treatment), Au@P-NPs showed higher insulin sensitivity and glucose tolerance in mice, which may be due to the elevated Ucp1 (uncoupling protein 1) expression in the brown adipose tissues and the mRNA expression of the membrane bile acid receptor TGR5. Additionally, Au@P-NPs reduced the *Lactobacillus* population and increased the *Akkermansia muciniphila* population in feces, while they also altered the plasma bile acid profile. Figure 5D shows the anti-obesity mechanism of Au@P-NPs, illustrating that the administration of Au@P-NPs increased energy expenditure and altered the bile acid profile via gut microbiota in HFD-induced obese mice for the subsequent conversion of white adipose tissues (WATs) into brown adipose tissues (BATs). More importantly, following 8 weeks of administration with Au@P-NPs, no toxic signs were shown in the liver, lung, kidney, heart, or pancreas, demonstrating the potential use of Au@P-NPs as alternatives to other anti-obesity drugs that are commercially available. Multiple meta-analyses also revealed that cinnamon possesses a positive effect on body mass index [133,134]. Based on seven meta-analyses, Keramati et al. [132] concluded that the intake of cinnamon can cause a significant reduction in body weight and body mass index. Based on these findings, cinnamon may be used as a drug for body weight reduction, or as a supplementary therapy in promoting body weight loss.

### 7.2. Cardiovascular Disease Protection

Oxidative stress and inflammation play critical roles in the development of cardiovascular diseases, with ROS being a vital risk factor for the former, and thus antioxidants capable of scavenging ROS can be considered as an effective strategy for the treatment of cardiovascular disease. On the other hand, TNF-α, a major proinflammatory cytokine inducing inflammatory responses in the vascular endothelium, has been reported to promote endothelial cell adhesion to circulating monocytes to encourage the elevation of the expression of intercellular adhesion molecule-1 (ICAM-1) and vascular cell adhesion molecule-1 (VCAM-1). To evaluate the efficacy of using CA in alleviating cardiovascular disease, Kim et al. [28] investigated the cytoprotective and anti-inflammatory effects of CA on H_2_O_2_-induced oxidative stress and TNF-α-exposed human umbilical vein endothelial cells (HUVECs). Both CA and methoxy-CA were shown to protect HUVECs against oxidative stress via the P38 pathway by activating nuclear factor erythroid 2 (Nrf2) nuclear translocation, resulting in a rise in heme oxygenase-1 (HO-1) expression. It is worth pointing out that Nrf2 is widely present in all human tissues, where it acts as a transcription factor and a regulator of the cellular levels of glutathione and thioredoxin, both of which are able to react with ROS and reactive nitrogen species. In addition, both CA and methoxy-CA markedly inhibited the adhesion of monocytic cells U937 to HUVECs by reducing the expression of VCAM-1 in TNF-α-exposed HUVECs. The anti-inflammatory effect was further confirmed by the attenuation of LPS-induced inflammatory cell infiltration in rats via the intraperitoneal injection of CA at 50 mg/kg BW.

**Figure 6:**
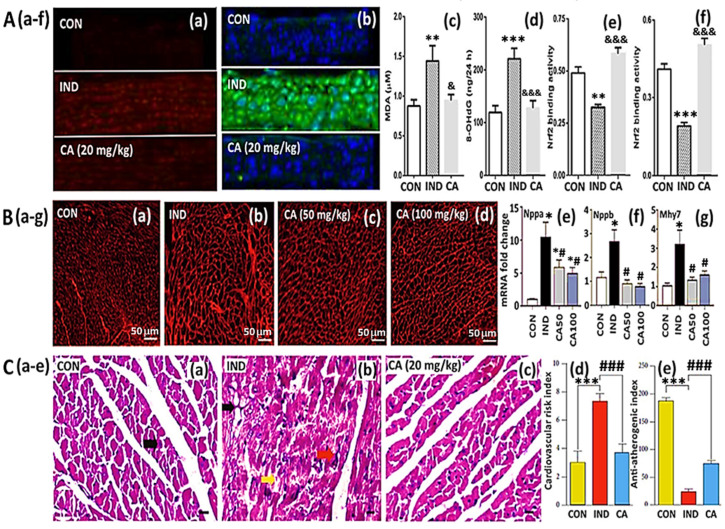
Cardiovascular-protective effects of cinnamon extract and cinnamaldehyde on animal models. (**A**(**a**–**f**)) Effects of CA on systemic and vascular oxidative stress in rats fed with sucrose (SU) and HFD compared to control, with dihydroethidium (DHE)-stained images depicting a significant reduction in DHE red fluorescence in aorta artery sections of SU/HFD-induced rats after treatment with CA (20 mg/kg BW) (**a**); nitrotyrosine staining images showing a color change from green to blue color in mesenteric sections of SU/HFD-induced rats after treatment with CA (20 mg/kg BW), (**b**) along with a decline in MDA levels in rat serum (**c**) and 8-OHDG levels in rat urine (**d**) as well as increases in Nrf2 expression in both the aorta (**e**) and mesenteric (**f**) arteries of rats. (**B**(**a**–**g**)) Representative wheat germ agglutinin (WGA)-stained images of frozen heart sections of control mice (**a**), phenylephrine-induced cardiac hypertrophy mice (**b**), cardiac hypertrophy mice treated with 50 mg/kg BW CA (**c**) and 100 mg/kg BW CA (**d**) along with a decrease in mRNA expressions including Nppa (**e**), Nppb (**f**) and Mhy7 (**g**). (**C**(**a**–**e**)) Hematoxylin–eosin-stained histological images of cardiac muscle sections of normal diet-fed rats (**a**) showing arranged cardiac muscle fibers with vesicular nuclei (indicated using arrows), HFD-induced rats (**b**) depicting degenerated cardiac muscle with severe degenerated cardiac muscle (yellow arrow), fragmented cardiomyocytes with some losing their nuclei (black arrow) and others showing pyknotic nuclei and mononuclear cellular infiltration (red arrow), as well as CA-treated HFD-induced rats (**c**) showing enhanced an architecture and integrity of the heart. Also, the effects of CA on the improvement of cardiovascular risk index (**d**) and anti-atherogenic index (**e**) in HFD-induced rats are shown. ** and *** in (**A**(**c**–**f**)) represent statistically significant data for induction group compared to control at *p* < 0.01 and *p* < 0.001 respectively. & and &&& in (**A**(**c**–**f**)) represent statistically significant data for CA treatment compared to control at *p* < 0.05 and *p* < 0.001 respectively. * and # in (**B**(**e**–**g**)) represent statistically significant data at *p* < 0.05 for CA treatment compared to control and induction group respectively. *** and ### in (**C**(**d**,**e**)) represent statistically significant data at *p* < 0.001 for CA treatment compared to control and induction group respectively. The scale bar in (**C**(**a**–**c**)) indicates 50 μm. BW, body weight; CON, control group; IND, induction group; HFD, high-fat diet; SU/HFD, sucrose and high-fat diet; CD, mice fed with normal chow diet; Nrf2, Nuclear factor erythroid 2-related factor 2; MDA, malondialdehyde; 8-OHDG, 8-hydroxy-2′-deoxygluanosine. Adapted with permission from Sena et al. [26], Ismail et al. [29] and Qian et al. [120].

In another study, vascular dysfunction caused by diabetes was reported to be alleviated by CA in diabetic mice with leptin receptor deficiency through the Nrf2 pathway. Leptin receptor deficiency can lead to severe obesity caused by frequent hunger and excessive eating [106]. Following the feeding of mice with a diet containing 0.02% CA for 12 weeks, ROS production was inhibited, while NO production was regulated, accompanied by an elevation in phosphorylated endothelial nitric oxide synthase (p-eNOS) and reductions in nitrotyrosine, P22 and P47 levels in mice aortas, compared to the control (Figure 6A). Also, the endothelium-dependent relaxation of aortas and mesenteric arteries in mice was improved by CA, implying that prolonged feeding with a CA-containing diet can protect against diabetic vascular dysfunction by inhibiting oxidative stress through the activation of Nrf2 and increases in the targeted genes HO-1 and quinone oxidoreductase-1 [119]. More recently, CA was also shown to ameliorate the endothelial dysfunction caused by oxidative stress and inflammation in diet-induced obese rats by activating Nrf2 [26]. After feeding rats with CA at 20 mg/kg BW/day for 8 weeks, the vascular oxidative stress, inflammation, and endothelial dysfunction in aortas and mesenteric arteries were significantly reduced, with a decline of MDA in rat serum and 8-hydroxy-2′-deoxyguanosine (8-OHDG) in rat urine compared to the control, as well as a concomitant rise in Nrf2, demonstrating again that CA is an effective activator of Nrf2 (Figure 6A).

Plaque buildup in arteries is a major factor responsible for cardiovascular disease. Although surgical procedures have been performed to restore artery size and blood flow, restenosis (the re-narrowing of blood vessels after treatment) can occur in patients due to the proliferation and migration of vascular smooth muscle cells (VSMC) and fibroblasts towards the inner vessel wall. Consequently, therapies to selectively inhibit VSMC proliferation and migration after surgical treatment need to be explored. In line with this objective, Cartaya et al. [88] compared the efficiency of CA and CA encapsulated into pluronic micelles in inhibiting VSMC proliferation and migration, as well as loading into ex vivo murine macrophage cells (RAW 264.7) and human macrophage cells (THP-1) for the subsequent alleviation of vascular injury. CA-pluronic micelles with a mean particle size of 235.8 nm, a polydispersity index of 0.190 and entrapment efficiency of 7.6% were prepared using a one-step direct dissolution assembly method. Both CA and CA-pluronic micelles reduced VSMC proliferation and migration, with EC_50_ values of 131 μM and 84 μM, respectively, accompanied by Nrf2 activation and antioxidant enzyme production (SOD and GSH). Compared to CA, CA-pluronic micelles were effectively internalized by macrophages in a time-dependent manner, leading to a reduction in nitrite production, with an enhanced accumulation on vascular smooth muscle cells within 12 h for subsequent Nrf2 activation and translocation into the nucleus within 24 h, aiding in the alleviation of vascular injury.

Myocardial hypertrophy is an adaptive response of the heart to a variety of pathological stimuli, with an increase in ventricular myocardial mass resulting in sudden cardiac death. Qian et al. [120] investigated the protective effects of TCA against cardiac hypertrophy in both in vitro and in vivo studies. In the in vitro study, cardiac hypertrophy induced with 50 μM phenylephrine in neonatal rat cardiomyocytes and treated with 5 μM TCA, TCA was found to suppress cardiac hypertrophy by decreasing the phosphorylation and nuclear localization of calcium/calmodulin-dependent protein kinase II (CaMKII) and extracellular signal-related kinase (ERK) in neonatal rat cardiomyocytes, while it blocked the hyperphosphorylation of ryanodine receptor type 2 and phospholamban, as well as restoring calcium handling and sarcomere shortening in adult mice cardiomyocytes. For the in vivo study, cardiac hypertrophy was induced by phenylephrine (75 mg/kg/day) with continuous stimulation for 2 weeks through a subcutaneously implanted mini-osmotic pump and the administration of TCA by oral gavage daily at a dose of 50 mg/kg BW or 100 mg/kg BW for the same period. Based on the morphological changes and echocardiographic parameters, as well as the expressions of hypertrophic genes and proteins, it was demonstrated that TCA at both doses could downregulate the phosphorylation of CaMKII and ERK through a decline in the expressions of hypertrophic genes (natriuretic peptide A (Nppa), natriuretic peptide B (Nppb) and myosin heavy chain 7 (Myh7)) and a thinning of interventricular septal thickness at both time points of systole ventricular contraction and diastole ventricular filling during the cardiac cycle, thereby alleviating cardiac hypertrophy in mice (Figure 6B).

Hyperlipidemia and dyslipidemia represent high blood cholesterol and an abnormal imbalance between good and bad cholesterol, respectively, resulting in increased risks of cardiovascular disease due to oxidative stress and inflammation. As CA has been shown to possess antioxidant and anti-inflammatory effects, Ismail et al. [29] explored the protective effects of CA against atherosclerosis induced by a high-fat diet orally administered to rats for 10 weeks, with CA at a dose of 20 mg/kg BW daily for the same period being shown to reduce the levels of serum total cholesterol and cardiovascular indices, with a concomitant rise in antiatherogenic index (Figure 6C). In addition, the levels of proinflammatory cytokines including IL-1β, IL-17, IL-6, and TNF-α were substantially reduced, along with an enhancement in antioxidant enzyme activities, including glutathione S-transferase, superoxide dismutase, catalase, and glutathione peroxidase, and a reduction in malondialdehyde, a major lipid oxidation product. Thus, the treatment of HFD-fed rats with CA may be protective against cardiovascular disease.

Recently, the anti-dyslipidemia and anti-platelet effects, as well as antioxidant activity, of *C. burmannii* bark extract were demonstrated in dyslipidemia mice by Sandhiutami et al. [121], showing that, following a dyslipidemia-inducing diet (80% egg yolk, 15% sucrose solution, and 5% animal fat) administered to mice for 14 days and then treated with cinnamon bark extract at 300, 400 and 500 mg/kg BW by oral gavage for 7 days, the total cholesterol was reduced respectively by 20.14%, 24.42%, and 35.76%, triglyceride by 4.09%, 8.74%, and 12.5%, and LDL by 38.17%, 53.8%, and 67.96%, while HDL rose by 27.29%, 67.8%, and 72.64%. In addition, the cinnamon bark extract showed an anti-platelet aggregation activity by prolonging bleeding time while increasing coagulation time and inhibiting adenosine diphosphate-induced platelet aggregation in mice serum.

### 7.3. Antidiabetic Activity

Diabetes is a metabolic disorder associated with blood insulin deficiency. Diabetes mellitus can be classified into type 1 diabetes mellitus (T1DM) and T2DM, with the former characterized by high blood glucose levels caused by insulin deficiency or a low sensitivity of the target organs to insulin secretion, and the latter characterized by persistent hyperlipidemia, hyperglycemia, and insulin resistance. The incidence of diabetes has significantly increased worldwide every year, with about 11% of Taiwan’s population, representing 2.3 million people, suffering from diabetes. Multiple kinds of hypoglycemic drugs with various mechanisms of action have been developed to maintain desirable blood glucose level. For instance, insulin sensitizers (Thiazolidinediones and Biguanides), insulin secretagogues (Meglitinide and Sulfonylureas derivatives), dipeptidyl peptidase-4 (DPP-4) inhibitors, α-glucosidase inhibitors, and exogenous insulin have been extensively used to treat diabetes mellitus. However, these traditional drugs lead to various adverse side effects. Alternatively, many medicinal plants represent excellent materials for treating diabetes, as they are safe and cost-effective, and the presence of phytochemicals such as flavonoids, carotenoids, alkaloids, saponins, terpenoids, and glycosides has been shown to contribute to the antidiabetic effect [122]. As a result, the World Health Organization (WHO) encourages the adoption of medicinal plant-based remedies to treat chronic diseases such as diabetes. Based on published reports, CA has become one of the main bioactive compounds for possible use in the treatment of diabetes.

**Figure 7:**
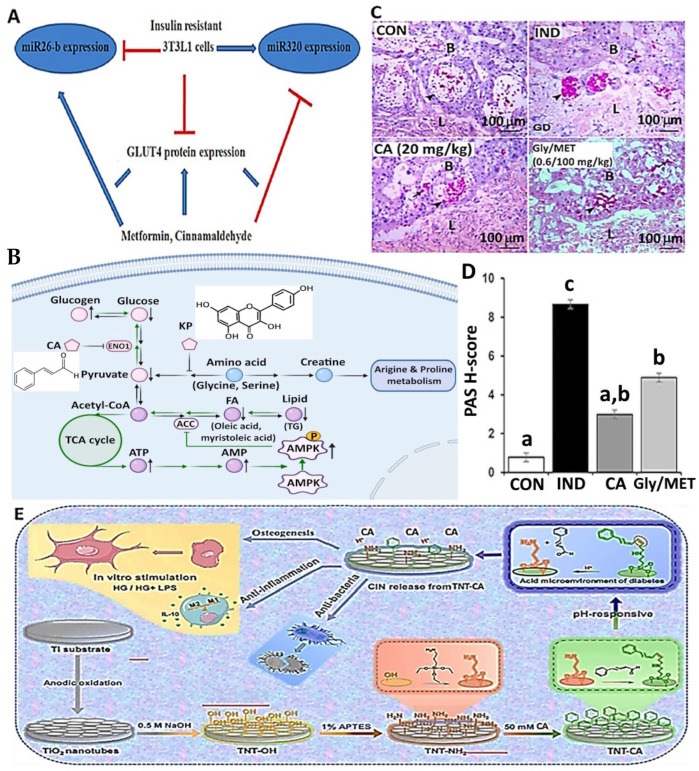
Antidiabetic mechanism of cinnamaldehyde in both in vitro and in vivo studies. (**A**) Probable mechanism of the effects of CA and metformin on insulin-resistant adipocytes (3T3L1 cells), with both increasing miR26-b expression with a concomitant reduction in miR320 expression through the GLUT4 dependent pathway. (**B**) Summarized mechanism of combination of CA and kaempferol for regulating glucose and lipid metabolism via AMPK in a diabetic mouse model, with green colored arrows indicating the pathways facilitated by their synergistic effect, while upward and downward arrows denote upregulation and downregulation, respectively. (**C**,**D**) Effects of CA and glyburide/metformin on gestational diabetes in fat–sucrose diet/streptozotocin-inducted rats with periodic acid-Schiff (PAS)-stained placenta images showing placenta glycogen content, showing several-fold increases in gestational placenta compared to control (**C**), along with a plot revealing a significant decline in PAS H-score for both CA and glyburide/metformin-treated gestational diabetic rats. The arrowheads in (**C**) represent PAS-positive glycogen storage cells, while normal arrows denote PAS-positive spongiotrophoblast cells. (**E**) A schematic diagram showing the fabrication of pH-responsive TNT-CA and its anti-inflammatory, osteogenic and antibacterial effects on a simulated diabetes condition using RAW 264.7 cells, BMSC cells and *Streptococcus mutans*/*Porphyromonas gingivalis* bacteria. Different letters (a–c) in (**D**) indicate statistically significant data at *p* < 0.05. B, basal layer; L, labyrinth layer; TNT-CA, titanium dioxide-cinnamaldehyde; HG/HG + LPS, high-glucose- or high-glucose plus lipopolysaccharide-induced BMSCs; BMSCs, bone marrow mesenchymal stromal cells; RAW 264.7 cells, macrophage cells; Ti, titanium; IL-10, interleukin-10; M1, iNOS marker (inducible nitric oxide synthase); M2, CD163 marker (cluster of differentiation 163); APTES, 3-aminopropyltriethoxysilane; ATP, adenosine triphosphate; AMP, adenosine monophosphate; AMPK, adenosine monophosphate kinase; FA, fatty acid; TG, triglyceride; ACC, acetyl-CoA carboxylase; TCA, tricarboxylic acid cycle; ENO1, enolase 1; KP, kaempferol; CA, cinnamaldehyde; PAS H-score, periodic acid-Schiff histochemical score. Adapted with permission from Naghiaee et al. [123], Gao et al. [125], Hosni et al. [122] and Lee et al. [131].

Insulin resistance is the major cause of T2DM, and some miRNAs that are directly linked with insulin resistance and related signals can act as biomarkers in evaluating the anti-diabetic effects of a drug or bioactive compound. In line with this hypothesis, Naghiaee et al. [123] evaluated the effects of CA-rich cinnamon extract on miRNA-320 and miRNA-26b expressions in insulin-resistant 3T3L1 adipocytes by comparing the results with metformin, a popular drug for the treatment of patients with type II diabetes. After culturing 3T3L1 preadipocytes in DMEM medium and their differentiation into corresponding adipocytes, the cells were initially stimulated for insulin resistance with high glucose (25 mM) and high insulin (1 μM), followed by treatment with cinnamon extract containing 100 μg/mL of CA and 10 mM of metformin alone or in combination. Compared to adipocytes without insulin resistance, the upregulated miRNA-320 and downregulated miRNA-26b, as well as GLUT4 expressions, in insulin-resistant 3T3L1 adipocytes were shown to be improved by all three treatments (CA-rich cinnamon extract, metformin, and their combination), suggesting that CA-rich cinnamon extract and metformin can restore the changes in miRNA expressions caused by insulin resistance, possibly via PI3K/Akt/GLUT4 or MAP kinase pathways (Figure 7A).

In another study, the CA standard was compared with metformin in terms of alleviating the metabolic syndrome caused by insulin resistance in rats fed with a high-fructose diet [124]. Initially, insulin resistance was induced in rats by feeding with a high-fructose diet for ten weeks, and the levels of glucose, insulin, glycosylated hemoglobin, HbA1c, total cholesterol, LDL-cholesterol, triglyceride, alanine transaminase (ALT), aspartate transaminase (AST), creatinine and uric acid increased in serum, accompanied by a decrease in glutathione and SOD levels. However, following the administration of CA at 40 mg/kg or metformin at 300 mg/kg by oral gavage daily for 4 weeks, the levels of the above biomarkers returned to normal, with both treatments showing comparable effects in alleviating metabolic syndromes caused by insulin resistance. A similar animal model was used more recently by Ghazal et al. [25] to demonstrate the ameliorating effect of CA, metformin and CA plus metformin on high-fat diet/streptozotocin-induced diabetes in rats. Prior to these treatments, the rats were treated with a high-fat diet for 4 weeks, and subsequently injected intraperitoneally with streptozotocin at 55 mg/kg in a citrate buffer (pH 4.4). After the fasting blood glucose levels reached >200 mg/dL, diabetic rats were administered orally by gastric tube with CA (40 mg/kg) and metformin (200 mg/kg) separately, as well as in combination, for 4 weeks. All three treatments exerted glucose and lipid-lowering effects by upregulating the mammalian target of rapamycin (mTOR) and mRNA-30a gene expressions and downregulating Beclin-1, LC3-II and Atg5 gene expressions in adipose tissue, with superior effects being shown in rats as a result of the combination treatment (CA plus metformin) compared to those treated with CA or metformin alone, implying that CA can be used as an adjuvant therapy with metformin to derive enhanced treatment efficiency, while minimizing the side-effects of metformin.

In addition to evaluating the combined effects of CA and the prescribed drug metformin for T2DM, the synergistic effects of CA with some other bioactive compounds were also investigated. For instance, Gao et al. [125] demonstrated that CA and kaempferol (a flavonol in edible plants) in combination at a dose ratio of 39:58 (mg/kg) could synergistically alleviate disorders associated with glucose and lipid metabolism in mice by lowering the levels of fasting blood glucose and HbA1c, total cholesterol, triglyceride and LDL cholesterol, while elevating both insulin and HDL cholesterol. Moreover, the combination of CA with kaempferol could significantly reduce the elevated ratio of adenosine monophosphate (AMP)/adenosine triphosphate (ATP) through the activation of 5′-adenosine monophosphate-activated protein kinase (AMPK), which was shown to be capable of inhibiting fatty acid synthesis and inducing catabolic lipid metabolism for the enhancement of the major energy-producing tricarboxylic acid (TCA) cycle (Figure 7B) [125].

As mentioned above, there is growing evidence showing that CA can lower blood glucose levels and improve streptozotocin (STZ)-induced rat diabetes. Celik et al. [126] explored the effect of CA on liver glutathione (GSH) and blood glucose levels in diabetic rats by using four treatments including control, CA, diabetes, and diabetes plus CA. It was shown that the blood glucose level in the group of diabetes plus CA decreased by 17.09% on the 30th day compared to the diabetes group, which may be related to increased insulin secretion and the peripheral use of glucose. In addition, the liver GSH level in the CA group (88.58 mmol/g) was much higher than in the diabetes plus CA group (38.67 mmol/g) and the diabetes group (37.56 mmol/g), revealing higher liver antioxidant activity for the CA group.

In another study, the treatment of diabetic rats with CA for 31 days was shown to reduce blood glucose by 1.33-fold and fasting insulin to a normal level compared to the diabetic group [127]. CA treatment also increased GSH content and elevated hepatic aortic eNOS, AKT2, and IRS1 mRNA expression levels by 14.95-, 13.21- and 6.93-fold, respectively, while it decreased the mRNA expression of aortic NADPH oxidase 4 (NOX4) to an optimal level in comparison to the diabetic group. AKT2 is a serine/threonine kinase that plays a role in the insulin signaling pathway, and is associated with insulin’s metabolic effects, while insulin receptor substrate 1 (IRS1) is the principal mediator of hepatic insulin action that maintains glucose homeostasis. Additionally, the potential anti-diabetes effects of CA may be due to the antioxidant activity, via the suppression of NOX4 expression, a major source of ROS in the heart, as well as the attenuation of serum advanced glycation end products (AGEs) and receptors of AGEs (RAGE). This outcome indicates the potent antioxidant activity of CA, which may make it helpful in the treatment of patients with T2DM [127]. It is worth pointing out that ACEs are lipids or proteins that become glycated as a result of exposure to sugars, and can be used as biomarkers in chronic diseases such as diabetes, atherosclerosis and Alzheimer’s disease. Similarly, CA administration was also shown to raise the expression levels of PI3K, IRS1 and AKT2 genes via the insulin signaling pathway [128]. It was also found that after CA administration, the eNOS (also known as nitric oxide synthase 3) expression in T1DM mice increased. Additionally, CA could protect pancreatic islet cells and enhance the expression of the insulin-regulated glucose transporter-4 (GLUT-4), found primarily in adipose tissues and striated muscle, aiding in the regulation of blood glucose levels while decreasing insulin resistance in T1DM mice [128].

The Wistar rats treated with essential oil nanoemulsions of *C. travancoricum* were found to display a significant reduction in blood glucose level on the 28th day, which may have been due to the increased glucose uptake and insulin secretion, as well as the inhibition of α-glucosidase and α-amylase activities [129]. In STZ-induced rats, the level of fructose-1,6-bis phosphatase rose following a decline in insulin. However, following treatment with *C. cassia*, the concentration of this enzyme declined, which may have been caused by a lower level of glucose in the blood from non-carbohydrate sources [130].

Gestational diabetes mellitus (GDM) is related to the impairment of the placental vascular structure, leading to the retardation of fetal growth. Specifically, GDM causes fetal and maternal hyperglycemia, placental dysfunction with subsequent fetal anemia, fetal hyperinsulinemia, a high serum erythropoietin level and hepatic iron deficiency, indicating fetal hypoxia. Recent research has evaluated the effects of CA (20 mg/kg/day), an antidiabetic agent used for GDM, and glyburide/metformin-HCl (0.6 + 100 mg/kg/day) as a reference drug, on the placental function of rats; only the CA treatment offered remarkable protection from GDM-related placental vasculopathy, aiding in the subsequent inhibition of fetal hypoxia, as shown in the periodic acid-Schiff (PAS) staining images (Figure 7C), along with a significant decline in the PAS H-score (Figure 7D). Apparently, CA is effective in the molecular regulation of metabolic activity, placental angiogenesis, and redox signaling. This finding further demonstrates that CA may provide a dual effect for the treatment of GDM during both fetal and maternal stages, via its antidiabetic effect and direct placental vasoprotective functions [122].

Following the administration of diabetic rats with a diet containing a nanoemulsion with a mean particle size of 36.58 nm prepared from cinnamon leaves for 30 days at 20 mg/kg, the glucose level in blood was reduced by 54.20%, which may have been due to the improvement of cell functions in the islets of Langerhans, enabling them to secrete more insulin and thus enhance glucose intake in cells. CA was also shown to inhibit the activities of aldose glucosidase and reductase, and also regulate the cell response of insulin, aiding in the enhancement of glucose metabolism via the activation of glycogen synthase and insulin receptor kinase [4].

The effects of selenium nanoparticles (SeNPS) and gum arabic (GA) in combination with COOE (*C. verum*, *Origanum majorana*, and *Origanum vulgare* extracts) on blood glucose reduction in diabetic zebrafish were studied by Gutierrez et al. [59]. Following treatment with COOE, GA-SeNPS, and GA-COOE-SeNPs at a dose of 20 µg/L and metformin at 20 mM, the blood glucose levels were remarkably decreased by 67.0%, 46.5%, 72.8%, and 64.33%, respectively. By comparison, GA-COOE-SeNPs possessed the highest antidiabetic activity, which may be due to the synergistic effect of Se nanoparticles, CA and phenolic compounds in COOE, which have been reported to be able to inhibit glucosidase activity and enhance glucose uptake.

Current research has shown that diabetes raises the risk of implanting failure, and thus the suitable surface remediation of dental implants for diabetic patients is vital. Lee et al. [131] synthesized a pH-responsive CA–titanium dioxide nanotube (TNT-CA) and reported that this fabrication possessed anti-inflammatory, osteogenic, and anti-bacterial effects under a simulated diabetes condition. This nanotube was fabricated by anodic oxidation, silylation, hydroxylation and Schiff base reaction for conjugation with CA, and its surface properties were analyzed. When the pH was 5.4, CA was released faster than than when the pH was > 7, as the amide bond was readily hydrolyzed at a lower pH (Figure 7E). This outcome confirms that TNT-CA exhibited a pH-responsive pattern, i.e., the acidic condition induced by diabetes may enhance CA release (Figure 7E). Comparatively, TNT-CA exhibited better osteogenic and anti-inflammatory activities as well as better bacterial resistance than titanium dioxide, with the difference in anti-diabetic activity probably being due to the difference in feeding period and dose, as well as the components used to prepare the nanosystems, especially for CA. This finding further demonstrates the significance of CA in improving diabetes.

### 7.4. Anti-Tyrosinase Activity

One of the most vital enzymes of the melanogenic metabolic pathway is tyrosinase, which is closely associated with malignant melanoma and many pigmentation disorders. It has been reported that tyrosinase overexpression can result in a high accumulation of melanin in the human body, leading to malignant melanoma and pigment spot diseases [135]. In the first step of the reaction, L-tyrosine is converted to 3,4-dihydroxyphenylalanine (L-DOPA) through the tyrosinase enzyme, followed by the conversion of L-DOPA to DOPA quinone, which is responsible for the darkening of the skin color. In the cosmetic industry, these enzyme inhibitors have attracted great attention because of their skin-whitening effects. Recently, many bioactive substances have been extracted and identified from *C. zeylanicum* for the evaluation of anti-tyrosinase activity. For example, Tepe and Ozaslan [136] analyzed essential oil composition by GC-MS; a total of 22 components were identified, with (E)-CA accounting for 81.39% and (E)-cinnamyl acetate (CAC) accounting for 4.20%. The anti-tyrosinase activities of CA and CAC were found to be 83.75% and 45.58%, respectively. However, with a CA-CAC ratio of 1:9, the antityrosinase activity was reduced to 36.26%, but it was raised to 86.85% with the CA-CAC ratio of 9:1. Apparently, CA is more important than CAC in decreasing tyrosinase activity. In a later study, Yu et al. [137] studied the anti-tyrosinase activity and mechanism of *C. cassia* Presl leaf hydrosol (CCPH) and *Cymbopogon citratus* Stapf leaf hydrosol (CCSH), with CA in CCPH and citral in CCSH accounting for 82.1% and 52.30%, respectively, following GC-MS analysis. Moreover, the IC_50_ values of CCPH and CCSH in the context of inhibiting tyrosinase activity were 84.8% and 23.9%, respectively, while the IC_50_ values of CA and citral were 53.3 mM and 21 mM, respectively. Collectively, CA and citral possessing aldehyde group and anti-tyrosinase activity may arise from the ability of carbonyl group to generate a Schiff base for reaction with a primary amine group through condensation and nucleophilic addition [138,139].

## 8. Amelioration of Neurological Disorders

Table 4 summarizes the effects of cinnamon extract, cinnamon oil and CA on various neurological disorders in both in vitro and animal models.

### 8.1. Alzheimer’s Disease

Alzheimer’s disease (AD) is characterized by memory impairment and cerebral pathological hallmarks, such as the accumulation of amyloid-beta (Aβ) plaques, neuronal loss, and the formation of neurofibrillary tangles in the hippocampus, amygdala and neocortex [158]. Recently, many studies have demonstrated that brain insulin deficiency and insulin resistance are the critical mediators of memory loss. A decline in the levels of insulin, insulin receptor substrate (IRS), and insulin receptor (IR) mRNA, all related to the pathway of phosphoinositide 3-kinase (PI3K)/serine/threonine-protein kinase (AKT), was shown in animals with AD [159,160]. This molecular pathway can target glycogen synthase kinase-3β (GSK-3β) as an important downstream element of this signaling pathway. More importantly, GSK-3β is a negative regulator of glucose homeostasis, inflammation, ER stress, mitochondrial dysfunction and apoptotic pathways. Tramutola et al. [159] further reported that GSK-3β plays a critical role in AD disorders caused by the deposition of Aβ plaques.

**Figure 8:**
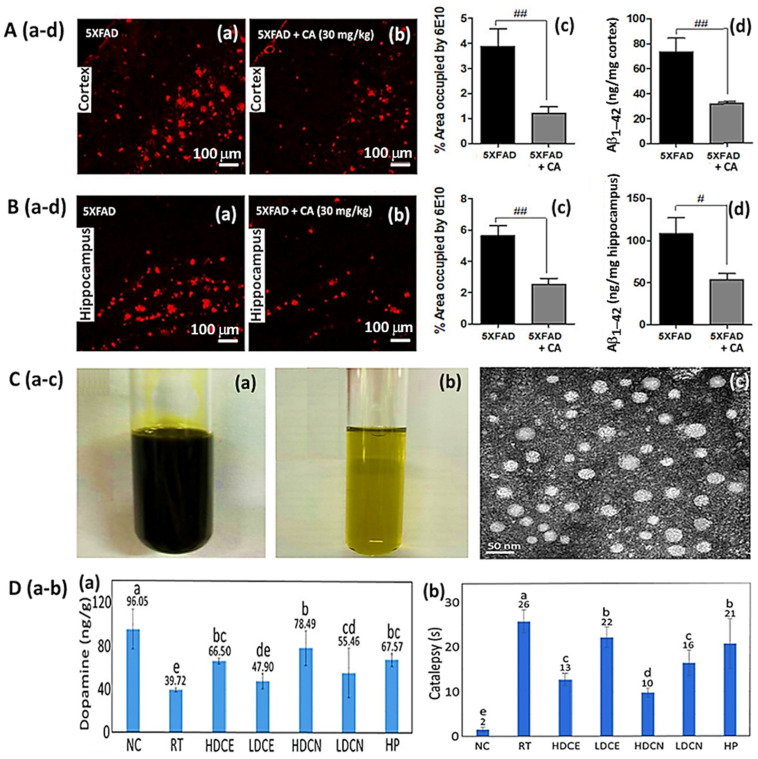
Effects of cinnamaldehyde and cinnamon extract on the amelioration of Alzheimer’s disease and Parkinson’s disease in animal models (**A**,**B**) and Parkinson’s disease in an in vitro model (**C**,**D**). (**A**(**a**–**d**)) Representative 6E10 staining images of Aβ deposition in the cortex of an AD mouse model (5XFAD) (**a**) and its mitigation after treatment with CA at 30 mg/kg (**b**) as well as the percentage of 6E10-positive areas in cortex (**c**) and Aβ1–42 levels in cortex (**d**). (**B**(**a**–**d**)) Representative 6E10 staining images of Aβ deposition in the hippocampus of an AD mouse model (5XFAD) (**a**) and its mitigation after treatment with CA at 30 mg/kg (**b**) as well as percentages of 6E10 antibody-positive areas in the hippocampus (**c**) and Aβ1–42 levels in the hippocampus (**d**). (**C**(**a**–**c**)) Appearance of cinnamon leaf nanoemulsion (**a**) and diluted cinnamon leaf nanoemulsion (**b**) as well as its transmission electron microscopic image with mean particle size of 30 nm (**c**). (**D**(**a**,**b**)) An increase in dopamine content (**a**) and improvement of catalepsy performance (**b**) in the striatum of rotenone-induced Parkinson’s disease rats following the daily administration of RT (2 mg/kg BW), RT (2 mg/kg BW) + HDCE (60 mg/kg BW), RT (2 mg/kg BW) +LDCE (20 mg/kg BW), RT (2 mg/kg BW) + HDCN (60 mg/kg BW), RT (2 mg/kg BW) + LDCN (20 mg/kg BW); HP (0.5 g/10 mL at a dose of 10 mL/kg BW) for 35 days. CON, control group; IND, induction group; CA, cinnamaldehyde; CE, cinnamon extract; BW, body weight; 5XFAD, AD mice model with severe amyloid pathology; Aβ, amyloid-beta plaques; NC, normal control without treatment induction and treatment; RT, rotenone dissolved in sunflower oil; HDCE, high-dose cinnamon extract; LDCE, low-dose cinnamon extract; HDCN, high-dose cinnamon nanoemulsion; LDCN, low-dose cinnamon nanoemulsion; HP, cinnamon powder in hydrosol. # and ## in both (**A**(**c**,**d**)) and (**B**(**c**,**d**)) represent statistically significant data for CA treatment compared to control. Data in (**D**(**a**,**b**)) are presented as mean ± standard deviation (*n* = 8) and data with different small letters (a–e) are significantly different at *p* < 0.05. Adapted with permission from Do et al. [143] and Wang et al. [5].

An important factor in Aβ generation is β-secretase activity, which can be used to decompose Aβ precursor protein. Do et al. [143] studied the underlying mechanism of TCA (30 mg/kg) in mitigating Aβ deposition in both the cortex and hippocampus of 5XFAD mice, and concluded that Aβ deposition was significantly decreased through a reduction in β-secretase activity and elevations in the mRNA and protein levels of three well-known regulators—β-secretase, peroxisome proliferator-activated receptor γ coactivator 1α, and silent information regulator 1 (Figure 8A,B). This outcome suggests that TCA may be a useful therapeutic drug in AD treatment, as it can reduce Aβ deposition by downregulating β-secretase expression via the activation of the regulators pathway. Kazerouni et al. [144] investigated the effect of CA at different doses on MAPK/ERK and hippocampal AKT alterations and memory impairment induced by scopolamine in mice. CA administration at 100 mg/kg was shown to remarkably inhibit the amnesic effect of scopolamine and the dysregulation of hippocampal AKT and MAPK. MAPK is a mitogen-activated protein kinase (serine/threonine-specific) responsible for the regulation of cell functions including proliferation, apoptosis, mitosis, gene expressions and differentiation, while ERK (extracellular signal-regulated kinase) can be involved in the regulation of mitosis, meiosis and postmitotic functions in differentiated cells. Most importantly, the MAPK/ERK pathway is a chain of proteins in the cells that communicate a signal from a receptor on the cell surface to DNA in the cell nucleus. Furthermore, the oral sub-chronic CA pretreatment possesses the ability to retard memory retrieval impairment induced by cholinergic blockade and restore hippocampal AKT and MAPK dysregulations. In another study, the protective effect of cinnamon extract (200 mg/kg BW/day) on aluminum neurotoxicity’s cerebellar pathology and behavior changes in Wistar rats with the AD model was investigated by Mustafa [142], demonstrating a significant reduction in glial fibrillary acidic protein expression in the cerebellar cortex with the enhanced protection of memory and learning ability.

Furthermore, CA at a dose of 100 mg/kg was demonstrated to improve recognition/spatial memory deficits and anxiety-like behavior through a reduction in hippocampal Aβ accumulation in an intracerebroventricular (ICV) streptozotocin (STZ)-induced rat model [30]. Western blot also indicated that the phosphorylation of hippocampal insulin receptor substrate 1 (IRS-1), AKT and GSK-3β was changed in this ICV-STZ rat model, with CA modulating this molecular pathway through an increase in the ratio of phosphoGSK-3βSer9/totalGSK-3β and phosphoAKTSer473/totalAKT and a reduction in the ratio of phosphoIRS-1Ser307/totalIRS-1. Also, the Morris maze test revealed that both control rats and CA (100 mg/kg)-treated STZ-induced rats displayed reduced thigmotactic behavior and enhanced focus on swimming to the resting platform quadrant, compared to the high thigmotaxis and erratic swimming in wrong quadrants shown by STZ-induced rats. In a later study, Sajadi et al. [140] investigated the effects of the ICV injection of insulin (5 mIU/5 μL) and the oral administration of cinnamon (200 mg/kg) extract on spatial memory and glucose transporter (GLUT) 1, 3, and 4 gene expressions in the hippocampus in an STZ-induced AD rat model; the behavior performance of rats in the Morris water maze test was improved in the group co-administered with CA extract and insulin injection, accompanied by the elevation of expressions of GLUT1, 3, and 4 genes in the hippocampal tissue. Apparently, a combination of cinnamon extract and insulin induces a synergistic effect on the regulation of dyslipidemia and the insulin signaling pathway.

In another study, Olorunnado et al. [141] explored the neuroprotective effects of trans-cinnamaldehyde (TCA) in the hippocampus of insulin-resistant rats, and demonstrated based on Y-maze and Morris water maze tests that TCA could alleviate the diabetes-induced impairment of learning and memory by reducing the levels of NF-κB and TNF-α in a rat hippocampus. Moreover, Tepe and Ozaslan [136] reported that two major compounds—TCA (81.39%) and cinnamyl acetate (4.20%)—of essential oil isolated from *Cinnamomum zeylanicum* (Blume) could ameliorate astrogliosis, pyknosis, and neurodegenerative symptoms in the hippocampus, in comparison with an untreated group, through the inhibition of cholinesterase and monoamine oxidase (MAO), an enzyme which can break down neurotransmitters such as dopamine, serotonin and norepinephrine, thus reducing Aβ accumulation in rat brains with AD. CA also showed great potential use in minimizing symptoms of AD through the inhibition of both the self- and the Cu^2+^-induced aggregation of Aβ1-42 by 57.78% and 84.53%, respectively, as well as MAO-A and MAO-B enzymes by 96.32% and 96.29% [136]. Momtaz et al. [161] reported that the presence of polyphenol in cinnamon extract could inhibit the formation, accumulation and toxic effects of Aβ plaques in PC12 neuronal cells. More specifically, cinnamon extract can interact with Aβ at the initial stage of self-aggregation via polyphenol entities to inhibit its aggregation and prevent Aβ toxicity, suggesting that these compounds may either cross the blood–brain barrier or could pass through other peripheral routes.

GABAergic (γ-aminobutyric acid) and glutamatergic are the two main synaptic types in the central nervous system, providing inhibitory and excitatory outputs, respectively, and a large body of data demonstrates that glutamatergic systems are often damaged when AD occurs [162]. To protect the glutamatergic components from damage caused by AD, Qian et al. [163] screened five bioactive compounds including propyl cinnamate, methyl cinnamate, procyanidin B1, procyanidin B2 and myristicin as the brain synapse-targeting active substances, identifying GABA pathway with γ-aminobutyric acid type A receptor subunit gamma 2 (GABRG2), GABA receptor alpha1 subunit (GABRA1), GABA receptor beta 2 subunit (GABRB2), and GABA receptor alpha 5 subunit (GABRA5) as the core therapeutic targets of cinnamon against AD-related GABAergic synaptic dysfunction. This finding suggests that the application of multiple components of cinnamon to multiple targets is promising in reducing the setbacks encountered by single-target and symptom-based strategies for drug discovery.

### 8.2. Parkinson’s Disease

Parkinson’s disease (PD), the second most common neurodegenerative disorder, is caused by the degeneration and loss of dopaminergic substantia nigra neurons in the midbrain [145]. The critical symptoms of PD include tremor, postural instability, rigidity, and a slowness of voluntary movement, which are mainly caused by the deficiency of dopamine and premature death of dopaminergic neurons in the nigrostriatal system [146]. The ultimate metabolite of 1-methyl-4-phenyl-1, 2, 3, 6 tetrahydropyridine (MPTP) is 1-methyl-4-phenylpyridinium (MPP^+^), which is responsible for the degeneration of nigrostriatal dopaminergic neurons [145]. The effects of *C. cassia*, *C. verum* and CA were evaluated against 6-hydroxydopamine (6-OHDA)-induced apoptosis in PC12 cells, an in vitro model of PD [145], with 6-OHOA being a synthetic neurotoxin that can destroy noradrenergic and dopaminergic neurons in the brain. Cell pretreatment with extracts and essential oils of cinnamon at a dose from 2.5 to 20 μg/mL and CA from 1.25 to 10 μM was found to inhibit ROS production significantly, and protect against 6-OHDA-induced cytotoxicity. In addition, the pretreatment of essential oils at 20 μg/mL could inhibit 6-OHDA-induced apoptosis, block the activation of the p44/42 pathway and reduce cytochrome-C expression in PC12 cells, resulting in the retardation of apoptosis and oxidative stress.

In a later study, Xu et al. [146] extracted cinnamon procyanidin oligomers (CPO-B) from cinnamon, and reported that it exerted a significant neuroprotection effect against MPP^+^-induced cytotoxicity in SH-SY5Y cells of PD disease. Also, CPO-B remarkably attenuated the effects of MPP^+^ and elevated the Bcl-2/Bax ratio to 78% compared to control cells, implying the anti-apoptotic role of CPO-B. Moreover, the upregulation of cleaved caspase-3 in the control treatment without CPO-B was reversed following treatment with CPO-B accompanied by a decline in the dephosphorylation of ERK1/2 caused by MPP^+^, suggesting the potential application of CPO-B in the treatment of PD.

Additionally, the insufficient intake of antioxidants may also cause the death of dopamine neurons. Wang et al. [5] prepared a cinnamon nanoemulsion with mean particle size, polydispersity index, zeta potential, and encapsulation efficiency values of 30.1 nm, 0.149, −43.1 mV and 91.6%, respectively (Figure 8C). In a PD rat model, this nanoemulsion, when applied at a dosage of 60 mg/kg BW, increased the tyrosine hydroxylase levels from 17.07% to 25.59%, dopamine contents from 17.08% to 49.39%, and activities of antioxidant enzymes including catalase from 8.56% to 16.94%, superoxide dismutase from 6.69% to 16.82%, and glutathione peroxidase from 2.09% to 16.94%, while decreasing the α-synuclein and malondialdehyde levels from 17.56% to 15.95% and from 22.47% to 15.47%, respectively (Figure 8D). Moreover, an improvement in catalepsy performance was demonstrated, with a high dose of nanoemulsion (60 mg/kg) being the most effective (Figure 8D). Obviously, the presence of major bioactive compounds such as CA should play a vital role in inhibiting ROS production in the possible treatment of PD. Collectively, the major bioactive compounds in cinnamon, such as CA, may exert neuroprotective effects in PD via several mechanisms, including mitochondria and cell apoptosis regulation, the elevation of antioxidant activity, anti-inflammation, reductions in α-synuclein aggregation, and the production of neurotrophic factors to support the growth, survival and differentiation of both developing and mature neurons in the central and peripheral nervous systems.

### 8.3. Ischemic Stroke

The top-ranked cause of disability and mortality worldwide is stroke. Currently, there are approximately 16 million stroke patients in the world, and recent reports show that 85% of all strokes are ischemic strokes caused by insufficient glucose and oxygen delivery and failure to maintain cellular hemostasis, with inflammation, excitotoxicity, apoptosis and oxidative stress being closely associated with stroke too [164]. One of the most famous traditional Chinese medicines is Guizchih-Fuling-Wan (GFW), which has inhibitory effects on ischemia/reperfusion (I/R)-induced brain damage. In an attempt to study protection against I/R-induced brain injury, mice were intraperitoneally administered with GFW at different doses and with herbs including *Paeonia lactifloa*, *Cinnamomi cortex*, *Prunus perisica*, *Paeonia suffruticosa* and *Poria cocos* before cerebral ischemia occurred [147]. Compared to the other groups, TCA in *Cinnamomi cortex* caused a significant reduction in the infarct area in cases of I/R-induced mice brain damage, and a reduced neurological deficit score. Also, TCA protects against I/R-induced brain damage by reducing neuronal apoptotic proteins, including NR2B, cytochrome-C, caspase 9, and caspase 3, by 95.00%, 96.23%, 96.23% and 93.07%, respectively. Under the same condition, the oral administration os GFW in a rat model before ischemia surgery, the percentages of reduction of the relative infarct area, COX-2 protein expression, and terminal deoxynucleotidyl transferase-mediated dUTP nick end labeling (TUNEL(+)) apoptosis followed a dose-dependent pattern, with values of 80.67%, 88.00% and 89.10%, respectively. This finding reveals that GFW is neuroprotective against I/R-induced brain damage, with TCA playing a major role in neuroprotection via modulation of the apoptosis pathway. Nevertheless, it should be noted that autophagy is closely related to apoptosis via different mechanisms. Evidence also shows that the mitochondria-related intrinsic apoptotic pathway activates caspase 3 for cell apoptosis execution, while the increases in autophagy and apoptosis suppression could lead to a recovery of I/R-induced brain damage in rodents [165].

Luo et al. [148] explored the effects and mechanisms of a combination of *Angelica sinensis* and *C. cassia* (AC) extracts on autophagic pathways using a rat permanent middle cerebral artery occlusion model. Following treatment with AC extract at 1.6, 3.2 and 6.5 g/kg, the scores of motor and sensor functions as well as ratios of glucose utilization in thalamic lesions were shown to improve in a dose-dependent manner, upregulating the expressions of Iba1, CD206, Beclin 1 and LC3 II while downregulating the expressions of TNF-α, IL-1β, IL-6, TLR4, phosphorylated-inhibitory kappa B kinase beta (IKKβ) and IκBα, nuclear P65, NLRP3, caspase-1, caspase-8 and cleaved caspase-3. Thus, the improvement of neurological function can be related to the suppression of NLRP3 inflammasome and the TLR4/NF-κB pathway and the enhancement of expressions of LC3-II and Beclin-1 after ischemic stroke. LC3-II can reflect starvation-induced autophagic activity, while Beclin-1 is a reliable regulator of autophagy, a process essential for mammalian survival. NLRP3 inflammasome is a critical component of the innate immune system that mediates caspase-1 activation and the secretion of proinflammatory cytokines IL1β/IL-18 in response to microbial infection and cellular damage. In addition, the production of ROS, mitochondrial dysfunction and lysosomal damage can trigger its activation. Similarly, the activation of TLR4/NFκB has been found to play a critical role in inflammatory diseases by controlling the expression of many cytokines such as IL-6 and TNF-α. Additionally, a combination of cinnamon and aspirin has been approved for use in the treatment of mild ischemic stroke or transient ischemic attack with 122 patients, including 62 in the aspirin–cinnamon group and 60 in the aspirin–placebo group, as a reduction in the risk of 90-day recurrent stroke and a decline in blood lipid, blood glucose, lipoprotein–related phospholipase A2, and high-sensitivity C-reactive protein levels, as well as a rise in HDL level, were observed compared with the aspirin–placebo group [149]. However, this clinical trial has limitations, and more large-scale clinical trials need to be performed in the future to verify the effectiveness of using cinnamon in the treatment of stroke.

### 8.4. Traumatic Brain Injury (TBI)

TBI occurs as a result of external force and may drastically affect brain function, leading to long-term disability and several pathogenic symptoms in brain tissue [151]. The severity of TBI is generally classified as mild, moderate or severe, with 70–80% of trauma patients with mild TBI also suffering from behavioral, cognitive and emotional problems [166,167]. The pathological mechanisms responsible for initiating and progressing TBI are divided into primary and secondary injury, with the former resulting from mechanical damage due to direct impact on neurovascular structures and glial cells, and the latter associated with inflammation, oxidative stress, excitotoxicity, energy deficiency due to ionic imbalance, and apoptosis [150]. Ionic imbalance can lead to transient osmotic gradients between neurons and glia and the extracellular space, thereby affecting neuronal activity strongly.

To explore promising alternative treatment methods for TBI, Bektasoglu et al. [150] studied the possible neuroprotective effects of CA on secondary brain injury following TBI induction by using a weight-drop rat model. The CA (100 mg/kg) group was found to lower myeloperoxidase activity as well as luminol-enhanced and lucigenin-enhanced chemiluminescence, revealing a reduction in inflammation and oxidative stress. In addition, CA could lower the histologic damage scores in the cerebral cortex and dentate gyrus of TBI rats, with the number of entries and spontaneous alternation percentage being reversed after CA treatment, as demonstrated in a Y maze test [150]. Apparently, CA enacts neuroprotective effects through the suppression of reactive oxygen species, leukocyte infiltration, and reactive oxygen metabolites’ generation, as well as limitations of neutrophil recruitment, resulting in improvements in acute hippocampal dysfunction and histologic damage. Likewise, cinnamon extract was shown to attenuate memory impairment and neuronal loss following TBI induction using a weight-drop mice model [151]. Specifically, less neuronal damage in the dentate gyrus and temporal cortex of the hippocampus was observed for mice receiving 10 μg/mL CA for 3 weeks, demonstrating the possible application of CA in the treatment of TBI patients. Nevertheless, the effects of CA-based nanosystems on TBI patients need to be further explored.

### 8.5. Multiple Sclerosis

Multiple sclerosis, an autoimmune disorder of the central nervous system (CNS), is caused by the targeting of the brain by immune cells, especially the myelin components, resulting in demyelination of axons and devastating associated symptoms [168]. This demyelinating pathology in multiple sclerosis may be attributed to inflammation, the loss of regulatory Tregs (T-cells), the hyperactivity of autoimmune Th1 and Th17 cells, the breakdown of the blood–brain barrier and the blood–spinal cord barrier, and the loss of neuroprotective molecules in the CNS [152]. Most importantly, T-cells have been demonstrated to play an important role in multiple sclerosis and its experimental autoimmune encephalomyelitis (EAES) in an animal model, with their deficiency in number and function being associated with the severity of multiple sclerosis [168]. Apparently, the therapeutic strategies for upregulating and maintaining T-cells can be beneficial for mitigating multiple sclerosis. For instance, Mondal and Pahan [152] have shown that *C. verum* is able to regulate the expression of myelin genes and suppress inflammation to prevent demyelination in CNS in a female PLP-TCR transgenic mice and adoptive transfer mouse model. Following the oral administration of cinnamon at a dose of 50 mg/kg BW/d, the symptoms of relapsing–remitting EAES were reduced, as only 40% of transgenic mice developed EAES. It is worth pointing out that both TH1 and TH2 cells are primarily involved in the regulation of humoral immunity, while TH17 cells represent an important and distinct subset of T helper cells that play a more critical role than Th1 cells in the progress of multiple sclerosis and EAES diseases. In EAES mice, cinnamon administration could suppress Th17 and Th1 responses while reinforcing Th2 response, blocking the disease progress of EAES. Consequently, the upregulation of Forkhead box protein 3 (Foxp3+) T cells may be necessary to the inhibition of the activation of autoimmune Th17 and Th1 cells, and for multiple sclerosis prevention.

In a later clinical trial, Delaviz et al. [153] investigated the effects of cinnamon on pain and anthropometric indices in patients with progressive–relapsing multiple sclerosis. Compared to the control group (500 mg wheat flour per capsule), a significant decrease in C-reactive protein and IL-6 mRNA levels (*p* < 0.05) was shown in the intervention group (500 mg cinnamon per capsule). Additionally, cinnamon showed a significant reducing effect in relation to the pain and body weight of patients with multiple sclerosis at week 8. However, cinnamon administration may not be responsible for the observed results due to the progressive nature of multiple sclerosis in patients, suggesting a need for further study to measure and compare various factors in different multiple sclerosis types.

### 8.6. Migraine

Globally, migraine is the third most prevalent disabling disorder and the most common type of primary headache, with growing evidence showing a close association between obesity and the high rate of incidence of migraine [154]. It has been reported that about 12% of the Western world’s population is affected by migraine, which is initiated in the central nervous system and implicated through neurovascular and metabolic changes in the brain caused by dysfunctional intracranial and extracranial blood vessels [169]. Studies have also shown that inflammation plays a pivotal role in causing pathological pain in migraine patients through the activation of nociceptive sensory neurons, with proinflammatory molecules such as calcitonin gene-related peptide, NO, IL-6 and TNF-α being associated with the underlying pathogenic mechanisms [155].

In a clinical trial study, Zareie et al. [155] studied the effect of cinnamon on migraine and inflammatory status via a randomized controlled trial. A total of 50 patients were randomized to receive three placebo capsules per day containing 100 mg of corn starch (control group) and three cinnamon powder capsules per day containing 600 mg of cinnamon for 2 months. The mean scores of frequency attacks as well as the serum NO and IL-6 levels were significantly reduced in the cinnamon group compared to the control group. More recently, they also evaluated the influence of *C. verum* on headache and anthropometric indices in 50 migraine patients using the same placebo and cinnamon dosages for 2 months [154]. Compared with the placebo group, cinnamon intake was found to inhibit weight gain, and increase body mass index and waist circumference, while reducing hip circumference and waist-to-hip ratio. Additionally, the reduction in headache daily result (HDR) was more pronounced in the cinnamon group compared with the placebo group, as evidenced by a decline of 118% in the former and 43% in the latter [154]. Thus, cinnamon may offer an alternative therapy for patients with moderate and severe migraines through the inhibition of pro-inflammatory cytokines. Nonetheless, a larger clinical trial with more patients should be conducted to verify the efficiency of treating migraines with CA and its nanoformulations.

### 8.7. Anti-Depression

Depression, a serious mental disorder characterized by slow response, persistent mood depression, lack of interest, sleep disorder, and low self-esteem, has become a serious social issue affecting people’s quality of life. Based on the WHO’s reports, around 322 million people are affected globally by depression, resulting in a risk of excess mortality or shorter life expectancy owing to suicides and accidents [156,170]. It is mainly caused by a deficiency of neurotransmitters such as serotonin, norepinephrine and dopamine, disturbances in neurotransmitter receptors, elevated levels of inflammatory cytokines, reduced levels of GABA, a hyperactivity of the hypothalamic–pituitary–adrenal axis, the impairment of the electrical activity of dorsal raphe neurons and a dysfunction of the brain–gut axis information conversion pathway [157]. Lin et al. [156] investigated the molecular mechanism of TCA on anti-depression in male BALB/c mice using a forced swimming test (FST). By comparison, TCA (50 mg/kg) showed a more pronounced effect in reducing immobility compared to the antidepressant drug fluoxetine (10 mg/kg), revealing that TCA improved depression-like behavior. In addition, TCA could elevate serotonin levels, accompanied by a decline in the ratio of Glu/GABA as well as a reduction in the levels of COX-2, cannabinoid receptor subtype 1 (CB1) and transient receptor potential vanilloid type 1 (TRPVI) in the mice hippocampus, leading to a subsequent improvement in depression through the involvement of both endocannabinoid and serotonin systems [156]. TRPV1, a Ca^2+^-permeable ion channel that can be activated by inflammation, is reported to be involved in the development of chronic pain and depression.

In a later study, Ma et al. [157] explored the effects of a cinnamon oil self-microemulsifying (CO-S-SME) drug delivery system on chronic unpredictable mild stress (CUMS)-induced depression-like behavior in mice. CO-S-SME (100 mg/kg) was shown to have a positive effect on the nervous system of mice through the elevation of neurotransmitters such as serotonin and a decrease in IL-6, TNF-α, and IL-1β levels, leading to an improvement in depression. It also reduced the levels of corticosterone by inhibiting the hyperactivity of the hypothalamic–pituitary–adrenal axis in CUMS mice. As the possible mode of application of CO-S-SME as an antidepressant is similar to the concept of using traditional Chinese medicine for the treatment of diseases involving multiple targets and pathways, it represents a promising approach that may be feasible for use in the clinical treatment of depression patients in the future. This novel drug delivery system was also shown to modify the intestinal flora composition by decreasing the ratio of *Firmicutes*/*Bacteroidetes*, reducing the relative abundance of *Lactobacillus* and modulating alpha and beta diversity of intestinal flora, leading to subsequent improvements in depression.

## 9. Attenuation of Bone and Joint Disorders

Table 5 summarizes the effects of cinnamon extract and CA on the alleviation of osteoarthritis, rheumatoid arthritis and osteroporosis in both in vitro and animal models.

### 9.1. Osteoarthritis and Rheumatoid Arthritis

Osteoarthritis (OA) is one of the most common degenerative musculoskeletal diseases involving chronic pain and dysfunction, and is characterized by the inflammation of the synovial membrane and the progressive destruction of articular cartilage. However, the treatment methods employed to reverse the progression of OA remain to be developed. Inflammation is regarded as an important factor in the pathogenesis of OA, with cartilage damage causing synovial inflammation and a chondrocyte-mediated inflammatory response. Thus, the efficient inhibition of chondrocyte inflammation may play a vital role in reversing the progression of OA. Chen et al. [171] explored the effects of CA on inflammation and cartilage degeneration in human OA chondrocytes obtained from patients who underwent total knee replacement surgery. Based on the cell counting kit-8 (CCK-8) assay, CA was shown to be non-toxic to OA chondrocytes at a concentration range of 10–50 μg/mL. Following pretreatment with CA at 10, 20 or 50 μg/mL for 24 h, OA chondrocytes were stimulated with LPS at a dose of 10 μg/mL, and the mRNA and proinflammatory cytokines including IL-1β, IL-6, TNF-α, MMP-13 and ADAMTS-5 (a disintegrin and metalloproteinase with thrombospondin motifs-5) were effectively inhibited in a dose-dependent manner compared to LPS-induced OA chondrocytes without CA treatment. In addition, CA at 20 and 50 μM was shown to suppress LPS-induced biomarkers of NF-κB such as p65 and IκB-α (inhibitory protein of NF-κB), demonstrating that CA may possess anti-inflammatory and chondroprotective effects through the NF-κB signaling pathway.

Furthermore, in a later study, the same research group demonstrated the involvement of ‘TLR signaling’ in inhibiting synovial inflammation [93]. More specifically, a total of 144 co-targeted genes for OA treatment with CA were collected from various databases and analyses of the Kyoto Encyclopedia of Genes and Genomes and Gene Ontology Functional Enrichment. These co-targeted genes were found to be mainly enriched in TLR, TNF and NF-κB signaling pathways, with molecular docking studies confirming the successful binding of CA with TLR2 and TLR4. Furthermore, the treatment of LPS (1 μg/mL)-induced human fibroblast-like synoviocytes with CA at 10, 20 and 50 µM could retard the synovial inflammatory responses including IL-1β, IL-6 and TNF-α through the blocking of the TLR4/MyD88 pathway. Similarly, Wu et al. [172] proved that TCA at 2, 5 and 10 µg/mL could inhibit the inflammation seen in IL-1β (10 ng/mL)-induced human knee articular chondrocytes via the phosphatidylinositol 3-kinase/protein kinase B (PI3K)/AKT pathway, as evidenced by a decline in levels of IL-8, prostaglandin E2 (PGE2), and TNF-α in a dose-dependent way, and a marked reduction in MMP-13, inducible nitric oxide synthase (iNOS), COX-2, and ADAMTS-5, as well as in the phosphorylation of AKT and PI3K, implying that TCA may be effective in protecting chondrocytes against OA through the attenuation of inflammatory response and the prevention of the degradation of the cartilage extracellular matrix.

**Figure 9:**
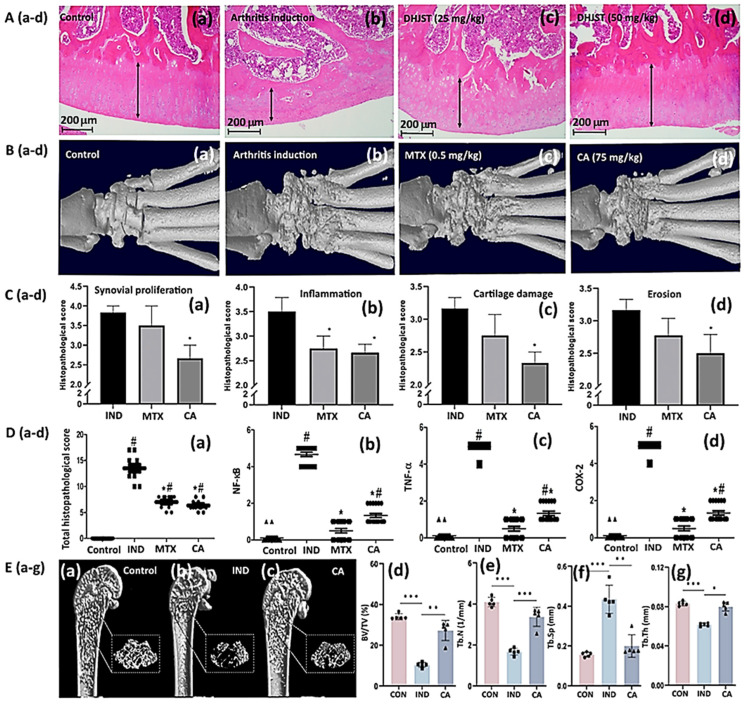
Effects of cinnamaldehyde and cinnamon-containing Chinese herbal formulation (DHJST) on the alleviation of osteoarthritis (**A**), rheumatoid arthritis (**B**–**D**) and osteoporosis (**E**) in animal models. (**A**(**a**–**d**)) Control rats (without osteoarthritis induction and treatment) (**a**), type II collagenase-induced osteoarthritis rats (**b**) and their histopathological changes after treatment with the cinnamon-containing Chinese herbal formulation (DHJST) at 25 mg/kg (**c**) and 50 mg/kg (**d**), with the double-headed arrow showing cartilage thickness. (**B**(**a**–**d**)) Microcomputed tomography scan bone images of control rats (**a**), type II collagenase-induced rheumatoid arthritis rats (**b**), MTX (0.5 mg/kg)-treated rheumatoid arthritis rats, and (**c**) CA (75 mg/kg)-treated rheumatoid arthritis rats (**d**). (**C**(**a**–**d**)) Histopathological scores of synovial proliferation (**a**), inflammation (**b**), cartilage damage (**c**) and erosion (**d**) for type II collagenase-induced, MTX (0.5 mg/kg)-treated and CA (75 mg/kg)-treated rheumatoid rat sections stained with hematoxylin–eosin. (**D**(**a**–**d**)) The levels of total histopathological (**a**) as well as NF-κB (**b**), TNF-α (**c**) and COX-2 (**d**) scores for control, CFA-induced, MTX (0.75 mg/kg)-treated and CA (100 mg/kg)-treated rheumatoid mice sections stained with hematoxylin–eosin. (**E**(**a**–**g**)) Microcomputed tomography images of mouse distal femur of control (**a**), ovariectomy-induced mice (**b**), CA (50 mg/kg)-treated ovariectomy mice (**c**) and the changes in trabecular bone microstructure such as BV/TV ratio (**d**), Tb.N (**e**), Tb.Sp (**f**) and Tb.Th (**g**). Double-headed arrow in (**A**(**a**–**d**)) indicate cartilage thickness. * in (**C**(**a**–**d**)) represent statistically significant data for positive control MTX or CA-treated group compared to control. * and # in (**D**(**a**–**d**)) represent statistically significant data for positive control MTX group and CA-treated group respectively compared to induction group at *p* < 0.05. *, ** and *** in (**E**(**d**–**g**)) represent statistically significant data at *p* < 0.05, *p* < 0.01 and *p* < 0.001 respectively. Dark circle, square and triangle symbols in (**D**(**a**–**d**)) and (**E**(**d**–**g**)) represent data points. CON, control group; IND, induction group; CA, cinnamaldehyde; MTX, methotrexate drug; CFA, complete Freund’s adjuvant; NF-κB, Nuclear factor kappa-light-chain-enhancer of activated B cells; TNF-α, tumor necrosis factor-alpha; COX-2, cyclooxygenase-2; BV/TV, bone volume/tissue volume ratio; Tb.N, trabecular bone plate number; Tb.Sp, trabecular bone spacing; Tb.Th, trabecular bone thickness. Adapted with permission from Tseng et al. [31], Cheng et al. [173], El-Tanbouly and Abdelrahman [174] and Lin et al. [176].

Recently, Tseng et al. [31] evaluated the anti-inflammatory effects seen in joints induced by the Chinese herbal formulation Du-Huo-Ji-Sheng-Tang (DHJST, a mixture of 15 herbs) and its simplified version Qi-tonifying (TH, a mixture of 4 herbs), with both containing *C. cassia* bark extract, using a cell model (LPS-induced RAW 264.7 cells and IL-1β-induced primary chondrocytes) and an animal model (monosodium iodoacetate-induced OA rats and type II collagenase-induced OA rats). Both treatments of DHJST (300 μg/mL) and TH (100 μg/mL) were shown to effectively inhibit expressions of NO, PGE2, iNOS and COX-2 in RAW 264.7 cells, while it was possible to downregulate iNOS and MMP-13 expressions in primary chondrocytes with DHJST treatment, as well as PGE2 expression in primary chondrocytes with TH treatment. For the animal model, OA rats induced with monosodium iodoacetate (50 mL of 80 mg/mL on day 0 and 50 mL of 40 mg/mL on day 6 in left ankle) were administered with DHJST or TH at a dose of 25 mg/kg BW and 50 mg/kg BW for 10 days, and showed a decrease in hind-limb weight-bearing, paw edema swelling and hot-plate latent pain response. For the evaluation of curative effects, DHJST or TH at the same dose was administered orally every day to OA rats induced with type II collagenase (4 mg/kg in left knee) for 21 days; the weight-bearing distribution in both hind limbs, as well as the low-grade inflammatory cell infiltration and cartilage thinning, were restored in a dose-dependent manner. Figure 9A illustrates the restoration of cartilage thickness in type II collagenase-induced OA rats after treatment with DHJST. These findings demonstrate that both DHJST and TH showed similar joint protection and curative effects in rats. Nevertheless, the chondroprotective effects, as induced by CA nanodispersions, need to be further investigated. More importantly, some more clinical trials should be conducted.

Several studies have also evaluated the efficacy of CA in alleviating rheumatoid arthritis (RA) in both in vitro and in vivo models. For instance, Cheng et al. [173] elucidated the molecular mechanism of CA in inhibiting the IL-1β-induced inflammation of human rheumatoid fibroblast-like synoviocyte cells MH7A when applied in three doses (40, 60 and 80 nM). CA was shown to downregulate the expressions of IL-6, IL-8 and TNF-α in MH7A cells, accompanied by impairments of Janus kinase 2 (JAK2), signal transducer/activator of transcription 1 (STAT1) and STAT3 signaling pathways by retarding the phosphorylation of JAK2, STAT1, and STAT3 without affecting the NF-kB pathway. When assessing rheumatoid arthritis progression with clinical scoring, as well as radiographic and histological examinations, collagen-induced arthritis rats administered with CA at a dose of 75 mg/kg BW daily by oral gavage for 21 days showed reduced swollen paw volume, joint swelling, bone erosion, destruction and arthritis severity, as well as reduced IL-6 levels in serum, compared to both control rats and collagen-induced arthritis rats administered orally with methotrexate (MTX), suggesting that CA could markedly ameliorate RA in rats to achieve enhanced joint protection. The microcomputed tomography scan images shown in Figure 9B reveal improvements in the bone erosion of collagen-induced RA rats after CA or MTX treatment, while Figure 9C shows a reduction in synovial proliferation, inflammation, cartilage damage and erosion for both CA- and MTX-treated RA rats. In a later study, El-Tanbouly and Abdelrahman [174] elaborated the protective mechanism of TCA in complete Freund’s adjuvant (CFA)-induced RA in mice given an intraperitoneal injection of 0.1 mL CFA in the first two days, and treated with TCA (100 mg/kg BW daily) or MTX (0.75 mg/kg BW, 3 times per week) for 3 weeks. A reduction in serum rheumatoid factor, arthritis index, paw swelling, cartilage and bone erosion, as well as in cytokines including TNF-α, IL-1β, IL-6, IL-23, IL-17 and COX-2, was observed through the modulation of the NF-κB pathway in inflamed paw tissues, with both TCA and MTX showing a similar inhibition effect. The scatter dot plots in Figure 9D depict declines in total histopathological score as well as NF-κB, TNF-α and COX-2 scores obtained from hematoxylin and eosin-stained sections of rat hind paw joints.

### 9.2. Osteoporosis

Osteoporosis (OP), a skeletal disorder caused by a decrease in total body bone mass, leads to the deterioration of bone microstructure with increased susceptibility to fragile fractures, disability and mortality [177]. Bone homeostasis is usually maintained by a balance between bone formation by osteoblasts and bone resorption by osteoclasts, which can be disrupted due to irregular diet, alcohol intake, smoking, aging and postmenopausal hormone deficiency in women [178]. Oxidative stress and mitochondrial dysfunction play a key role in the OP microenvironment, reducing the survival of bone marrow mesenchymal stromal cells (BMSCs), resultinsg in a decrease in their osteogenic ability [176]. Current therapies for OP including bisphosphonates, denosumab, calcitonin, fluoride, hormone replacement therapy and selective estrogen receptor modulators have been reported to cause side effects including fatty liver, nausea, headache, limb pain and breast cancer [179]. Thus, it is pivotal to find a replacement for drugs that can treat OP without side effects.

Hong et al. [175] evaluated the effects of CA on OP in both preosteoblast cells MC3T3-E1 and ovariectomized mice, demonstrating its efficiency in inducing osteoblast differentiation and elevating osteogenic markers to recover the bone loss caused by OP. Following the treatment of MC3T3-E1 cells with CA at 25, 50 and 100 μM, both alkaline phosphatase and calcium deposit, which are markers of new bone formation, were shown to increase in a dose-dependent manner, accompanied by a rise in protein expressions including transforming growth factor beta (TGFβ), bone morphogenetic protein 2/4 (BMP2/4) and phospho-suppressor of mothers against decapentaplegic 2/3 (pSmad2/3), as well as mRNA expressions osteocalcin and runt-related transcription factor 2 (Runx2). In the in vivo study, after performing ovariectomies on mice by removing the bilateral ovaries, they were fed orally with 5, 10 and 20 mg/kg BW of CA 5 times a week for a total of 10 weeks, and it was demonstrated that several bone indices (bone mineral density, volume and surface area; trabecular thickness; spacing and plate number; cortical bone thickness) increased, with a concomitant rise in osteocalcin and procollagen type 1 levels in mice serum, implying that CA could ameliorate postmenopausal OP through the BMP/TGFβ/Smad signaling pathway.

In a later study, the ability of CA to restore bone homeostasis and bone loss by alleviating oxidative stress and mitochondrial damage induced by H_2_O_2_ in the BMSCs and ovariectomized mice model was studied by Lin et al. [176]. The treatment of BMSCs isolated from the femurs and tibias of mice with CA at 50 and 100 μM could dose-dependently reduce the apoptosis of BMSCs induced by H_2_O_2_ by increasing Bcl-2 and decreasing the levels of Bax, caspase-3 and cytochrome-C. CA was also shown to scavenge ROS to alleviate oxidative stress and elevate optic atrophy 1 (OPA1, a mitochondrial inner membrane fusion protein), as well as decrease dynamic-related protein (Drp1, mitochondrial fission protein) to mitigate mitochondrial dysfunction in H_2_O_2_-induced BMSCs. Moreover, both osteogenic differentiation and mineralization were partially restored via the Nrf2/heme oxygenase-1 (HO-1) pathway by increasing the levels of osteogenic transcription factors including collagen type 1 alpha 1 chain (COL1A1), Runx2 and osteocalcin (OCN), as well as Nrf2 and HO-1, in H_2_O_2_-induced BMSCs. In the in vivo study, the intragastric administration of CA (at 50 mg/kg BW per day for 8 weeks) to ovariectomy-induced mice was shown to partially restore oxidative stress and bone loss by ameliorating bone trabecular microstructures via increasing trabecular bone volume/tissue volume ratio, plate number and thickness, decreasing trabecular bone spacing, and elevating the expressions of Nrf2, Runx2 and HO-1 in the distal femurs of mice (Figure 9E).

More recently, Ji et al. [32] demonstrated the inhibitory effect of CA on the osteoclast activity in streptozotocin-induced diabetic OP rats through the modulation of netrin-1/deleted in colorectal cancer (DCC)/uncoordinated 5 netrin receptor B (UNC5B) signal transduction. Following the intraperitoneal injection of streptozotocin at 60 mg/kg BW into Sprague–Dawley (SD) rats, diabetes-induced OP rats treated with CA at 20 and 40 mg/kg BW daily for 12 weeks showed increases in bone strength and bone remodeling activity, as well as improved bone structure, by increasing bone volume/tissue volume ratio, trabecular plate number and thickness, as well as decreasing bone surface area/bone volume ratio and trabecular spacing. Also, the levels of netrin-1, DCC, UNC5B, receptor activator of nuclear factor kappa B ligand (RANKL) and osteoprotegerin (OPG) were increased, while TGF-β, cathepsin K, tartrate-resistant acid phosphatase (TRAP) and the receptor activator of nuclear factor kappa B (RANK) levels were suppressed in diabetes-induced OP rats in a dose-dependent manner, indicating that the osteoclast activity induced in diabetic rats can be inhibited by CA. Nevertheless, the efficiency of treating OP with nanoformulations of CA and clinical trials should be assessed in the future.

## 10. Conclusions and Future Perspectives

This review article summarizes the recent advances made in the extraction and analysis of the release behaviors, pharmacokinetics, and biological functions of cinnamon extract/oil, CA, and its derivatives. Many studies have demonstrated the improved efficiency of CA and its derivatives in alleviating chronic diseases. Moreover, compared with CA and its derivatives, conjugates prepared with polymers, biomolecules and drugs, as well as their micro/nanoencapsulated forms, were shown to exert higher biological activities, suggesting a possible synergistic effect. Future studies should focus on evaluating the emerging extraction techniques for possible industrial scale-up along with the development of green and sustainable methods for enhancing the extraction efficiency and reducing capital costs, time costs, energy requirements and harmful solvents used. Also, a more sensitive, robust, rapid and sustainable technique for the analysis of CA and its derivatives is needed. Some more comprehensive in vivo studies, especially clinical trials, dealing with the elucidation of the molecular mechanism in CA and its derivatives when ameliorating a specific disease, should be undertaken to provide a detailed structure–activity relationship. Additionally, a more systematic evaluation of the toxicity associated with conjugates and micro/nanoencapsulated forms of CA and its derivatives is necessary. Most importantly, increased focus should be placed on clinical trials to transform the outcome of in vivo studies from bench to bedside. Regarding future applications, the most important is in the treatment of diseases associated with memory loss, especially given the high prevalence and incidence rates of AD and Parkinson’s disease worldwide, as currently there are no commercial drugs available that can offer a cure.

## Figures and Tables

**Table 1:** Analytical methods used recently for the determination of cinnamaldehyde and other compounds in cinnamon bark and leaf.

Cinnamon Part	Extraction	Methods	Analysis Condition	Analytical Performance	Reference
CE bark	Ultrasound-assisted extraction (10 g of dried sample was refluxed with 100 mL methanol for 1 h)	HPTLC	Glass-backed plates precoated with NP silica gel 60 F254S plates (10 × 20 cm); mobile phase of cyclohexane/ethyl acetate (90:10, *v*/*v*); detection wavelength: 296 nm	Separation of 2 compounds within 32 min; LOD: 3.56 ng/band; LOQ: 10.68 ng/band; Recovery: 98.45–101.16%; CV: 0.52–0.88%	Foudah et al. [56]
CE bark	Ultrasonic-assisted extraction (200 mg sample was sonicated with 5 mL methanol for 30 min at 25 °C)	HPLC	C18 column (150 × 4.6 mm, 5 μm); isocratic mobile phase of acetonitrile and 0.04% acetic acid at a ratio of 60:40 (*v*/*v*); detection wavelength at 280 nm; flow rate at 1.0 mL/min; column temperature at 29 °C	Separation of CA within 20 min; LOD: 0.069 ppm; LOQ: 0.230 ppm; Recovery: 97.94–104.69%; Precision RSD: 0.92–2.55%	Puspita et al. [57]
CE bark	Ethanolic extracts prepared using 10 mL of ethanol for 1 g solid mass at 37 °C	RP-HPLC	C18 column; gradient mobile phase of water and acetonitrile at a ratio of 40:60 (*v*/*v*); detection wavelength at 280 nm; flow rate at 0.8 mL/min; column temperature at 40 °C	Separation of CA with yield at 3.05 mg/g	Othman et al. [58]
CE leaf	Ultrasonic-Assisted Extraction (1 g of sample was sonicated with 40 mL ethanol for 2 h at 60 °C)	UPLC-MS/MS	C18 100 Å LC column (100 × 2.1 mm ID, 1.6 μm particle size); gradient mobile phase of 0.025% acetic acid in water and 0.025% acetic acid in methanol; flow rate at 0.3 mL/min; column temperature at 30 °C; multiple reaction monitoring (MRM) mode in MS/MS detection	Separation of 15 compounds within 14 min; LOD: 0.08–8.40 ng/g; LOQ: 0.24–25.19 ng/g; Inter-day RSD: 1.81–8.93% Intra-day RSD: 1.93–8.21%	Huang and Chen [4]
CE leaf	Ultrasonic-assisted extraction (1 g of sample was sonicated with 30 mL ethanol for 2 h at 60 °C)	UPLC-MS/MS	C18 100 Å LC column (100 × 2.1 mm ID, 1.6 μm particle size); gradient mobile phase of acetic acid in water and 0.025% acetic acid in methanol with a ratio at 40:60, column temperature at 30 °C; flow rate at 0.3 mL/min; MRM mode in MS/MS detection	Separation of 15 compounds within 14 min	Wang et al. [5]
CE leaf	Ultrasound-assisted extraction (10 g sample sonicated with 66% ethanol for 29 min)	LC-MS/MS	C18 analytical column (2.1 × 100 mm ID, 3.5 µm particle size); mobile phase: 0.1% formic acid in water and water–acetonitrile	Separation of 12 compounds within 42 min	Gutierrez et al. [59]
CE bark	Steam distillation (60 g sample soaked in 300 mL 50% aqueous ethanol for 24 h and evaporated)	GC-MS	5MS-HP column (30 m × 0.025 mm ID, 0.25 μm film thickness); nitrogen as carrier gas; column temperature programmed from 45–250 °C at 5 °C/min	Separation of 19 compounds within 46 min	Emami et al. [60]
CE bark	Microwave-assisted extraction (ethanol as a solvent to solid ratio of 1:6 for 30 min)	GC-MS	__ ^a^	Separation of 5 compounds within 30 min	Fazillah et al. [61]
CE leaf	Steam distillation ^b^	GC-MS	Capillary column (HP-5MS UI, 30 m × 0.25 mm ID, 0.25 μm film thickness); helium as carrier gas, column temperature programmed from 120 to 260 °C at 10 °C/min	Separation of 10 compounds within 25 min	Yitbarek et al. [62]
CE leaf	Hydrosol (100 g of sample was soaked in deionized water for 30 min followed by hydrodistillation for 120 min)	GC-MS	HP-5MS columns (60 m × 0.25 mm ID, 0.25 μm film thickness); helium as carrier gas; flow rate at 1 mL/min; column temperature programmed from 50 to 300 °C at 10 °C/min	Separation of 12 compounds within 60 min	Yu et al. [52]
CE bark	Hydrodistillation ^b^	GC-MS; GC-FID	GC-MS: HP5MS column (30 m × 0.25 mm ID, 0.25 mm film thickness); helium as carrier gas; flow rate at 1.2 mL/min	Separation of 3 compounds within 24 min	Rehman et al. [63]

^a^ The GC-MS conditions are not specified in the article published by Fazillah et al. [61]. ^b^ The conditions for steam distillation and hydrodistillation are not specified in the articles published by Yitbarek et al. [62] and Rehman et al. [63], respectively. CE, cinnamon extract; CA, cinnamaldehyde; HPTLC, high-performance thin-layer chromatography; HPLC, high-performance liquid chromatography; RSD, relative standard deviation; LOD, limit of detection; LOQ, limit of quantitation; CV, coefficient of variation; RP-HPLC, reversed-phase high-performance liquid chromatography; UPLC, ultra-high-performance liquid chromatography; GC-MS, gas chromatography–mass spectrometry; LC-MS/MS, liquid chromatography with tandem mass spectrometry; GC/FID, gas chromatography–flame ionization detector; ID, internal diameter; MRM, multiple reaction monitoring; NP, normal phase.

**Table 2:** Effects of cinnamon extract and cinnamaldehyde on various cancer cells and tumors in an animal model.

Sample/(CA/CE)	Cancer Cell/Animal Model	IC50; Dose	Inhibition Mechanism	Outcome	Reference
CE and CA	Oral cancer cells (SCC-4, SCC-9, SCC-25)	IC50: 30–100 μg/mL (CE), 30–250 μM (CA) CE dose: 0–400 μg/mL CA dose: 0–960 µM	↓ PI3k-AKT-mTOR pathway related to VEGF, COX-2, Bcl-2, NF-κB, and proteins	Both CE and CA inhibited the invasion and cytoplasmic translocation of NF-κB in these cell lines	Aggarwal et al. [94]
CA	Breast cancer cells (MDAMB-231)	IC50: 12.23 μg/mL Dose: 15 and 20 μg/mL	↓ PI3K-AKT, peroxisome proliferator-activated receptor pathway	CA inhibited cell proliferation, migration and invasion ability, as well as promoting cell apoptosis	Liu et al. [95]
CA	Prostate cancer-associated fibroblasts	IC50: 74.66 μΜ Dose: 32–150 µM	↑ Cytochrome c, Bax, caspase 3, PARP; ↓ Bcl-2, caspase 9, DEF-45	CA induced cell apoptosis by ROS generation and decreases in mitochondria membrane potential	Han et al. [96]
CA	Bladder cancer cell (5637)	Dose: 0.02–0.08 mg/mL	↓ ErbB2, HSF1, and LDHA, protein level of HSF1 and LDHA, LDH activity; ↓ cell migration, glucose consumption, and lactate production	CA induced apoptosis and decreased cell growth by reducing ErbB2-HSF1- LDHA pathway	Aminzadeh et al. [97]
CA, DOX and CA-DOX	Glioblastoma cells (U87MG)	IC50: 11.6 µg/mL (CA), 5 µg/mL (DOX)	↑ Bcl-2, Bax, caspase-3, caspase-9 and cell population subG1 phase ↓ glutathione S-transferase, ATPases, ΔMψ potential	CA enhanced the apoptosis induced by DOX	Abbasi et al. [98]
CA + hyperthermia	Lung cancer cell (A549)	Dose: 150 and 200 µM	↑ ROS, MAPK phosphorylation and HSP70 ↓ VEGF, cyclin D1, MMP-9, MMP-2	A combination of CA with hyperthermia treatment effectively inhibited lung cancer cells through ROS generation	Park et al. [99]
CA-poly(thioacetal) NPs	Colon cancer cells (CT26)	Dose: 10–60 μg/mL	↑ ROS, ↓ mitochondrial membrane potential	Endogenous ROS induced cleavage of polymer to release CA for generation of ROS through mitochondrial dysfunction.	Tu et al. [100]
RSL3@PCA	Breast cancer cells (4T1)	Dose: 0.25–15 μM	↓ lipid peroxides in cells, intracellular GSH and GPX4 activity	CA and RSL3 induced ferroptosis by reducing lipid peroxides to inhibit cancer cell growth without affecting normal cell/tissue	Yan et al. [101]
MON CA-TPP @HA	Breast cancer cells (4T1)	Dose: 0–100 μg/mL	↑ ROS resulting in enhanced oxidative stress and immunogenic cell death	HA help to target cancer cells and TPP enable binding with mitochondria, while overexpressed GSH in cancer cells cleave the disulfide bond in MON for CA release and ROS generation	Zhu et al. [102]
ETS@PCA	Breast cancer cell 4T1 (1 × 10^6^)-induced tumor in BALB/c mice	Dose: 5 mg/kg; IV	↑ ROS ↓ GSH, topoisomerase II	These self-assembled NPs responded to an acidic tumor environment by releasing CA for the rapid depletion of GSH, contributing to enhanced antitumor efficiency	Wu et al. [103]
HA-CD/FC-CA NPs	Breast cancer cell (MCF-7), 4T1 and NIH/3T3 cells	Dose: 2.5–250 μM	↑ ROS, H_2_O_2_, Fenton reaction, OH^∙^ radical	CA induced H_2_O_2_ levels for reaction with ferrocene through cascaded Fenton reaction to generate cytotoxic hydroxyl radicals for killing cancer cell	Xu et al. [104]
HA-CD/FC-CA NPs	Female BALB/c mice bearing 4T1 tumor xenograft model	Dose: 7 mg/kg FC+ 4 mg/kg CA; IV	↓ tumor weight	An in vivo study confirmed the cascading Fenton reaction for excellent anti-tumor effects	Xu et al. [104]
CA/DATS@PLGA-PEG NPs	Breast cancer cells (MCF-7)	Dose: 100 mM (CA) + 50 mM (DATS)	↑ ROS, ↓ GSH	Combined killing of cancer cells by increasing oxidative stress by CA and depleting GSH by DATS	Liu et al. [105]
TCA- CMPs	Human epithelial cervical cancer (HeLa)	Dose: 0–50 μM	A cumulative TCA release rate was higher at pH 6.5 (tumor environment) than at pH 7.4 (neutral) with 77.9% cytotoxicity being shown after 48 h	CMPs facilitated CA release in the tumor environment for enhanced uptake and inhibition of cancer cell growth	Barrera-Martinez et al. [106]
B-C-CF-CA NPs	Lung cancer cells (A549)	IC50: 2 μg/mL Dose: 7.8–500 μg/mL	Cytotoxicity through diffusion-controlled CA release with an 18-fold reduction in IC_50_ compared to only CA	Exhibited acid-responsive CA release for enhanced targeting with calcium ferrite enabling magnetically modulated faster CA delivery	Purushothaman et al. [107]
DOX-CLC NPs	Human liver carcinoma (Hep-G2)	CA Dose: 1–16 μg/mL DOX dose: 1–16 μg/mL	↑ Mitochondrial permeability transition, ROS production, caspase activation, apoptosis	LA in DOX-CLC-NPs facilitated high cellular uptake, while CA increased ROS production and DOX promoted direct cytotoxicity for synergistic growth inhibition	Zhou et al. [108]
DOX-CLC NPs	H22 cells (1 × 10^6^)-injected into left armpit of ICR male mice	CA dose: 6 mg/kg; DOX dose: 6 mg/kg; IV	↓ Tumor weight and size	DOX-CLC NPs were passively enriched in tumor tissues through the EPR effect with increased cellular uptake and release of DOX and CA, exhibiting a synergistic anti-tumor effect	Zhou et al. [108]
CA polymeric micelles	Colon cancer cells (SW620)	Dose: 50–300 μM	↑ Oxidative stress	Higher cytotoxicity due to the pH-induced cleavage of imine linkages under acidic conditions resulting in polymeric micelle disassembly and the release of CA	Han et al. [109]
CA-FU NPs	Hepatocellular cells (H22)	IC50: 21.291 μg/mL Dose: 3.85–385 µM	↑ Release at pH 5 than 7, cytotoxicity	Apoptosis by cellular uptake and cleavage of acetal and ester bond for enhanced apoptosis	Fang et al. [23]
CA-FU NPs	Injection of H22 cells (1 × 10^6^) into the back of male ICR mice	Dose: 7.65 mg/kg; IV	↓ Tumor weight and size with CA-5FU showing higher tumor growth inhibition compared to CA or 5FU	CA-FU induced significant tumor apoptosis or tissue necrosis through oxidative damage by CA and anti-metabolic effects by FU, combining into a synergistic effect.	Fang et al. [23]
BSA-CA NPs	Laryngeal squamous cell (Hep2), lung cancer (A549), hepatoma cancer cell (HepG2), malignant melanoma cell (A375)	Dose: 100–700 μg/mL	>50% inhibition towards A375, A549 and HepG2 cells, and >80% towards Hep2	BSA–CA NPs promised anticancer activity against various cancer cells with higher inhibition efficiency compared to Hep2 cells	Chang et al. [110]
CA and/or tannic acid- gelled-oil NPs	Breast cancer cell (MCF-7) and Lung cancer cell (A549)	EC50: 107.06 μg/mL (CA-TA) and 223.11 (CA) μg/mL Dose: 1.6–1000 μg/mL	81.7% and 57.5% cytotoxicity towards MCF-7 and A549 cells	Enhanced antioxidant and anticancer activity through encapsulation of a mixture of bioactive natural compounds	Asadi-Yousefabad et al. [111]
CA-IONPs/FA	Breast cancer cells (MCF7, MDAMB231)	IC50: 2.84 μg/mL (MCF 7), 17.44 μg/mL (MDAMB231) CA dose: 0.312–1.25 μg/mL	↑ Mitochondrial depolarization, calcium release, caspase expression	Enhanced cellular uptake and localization in both cytoplasm and nucleus promoted apoptosis	Shetty et al. [24]
CA-IONPs/FA	E0771 (1 × 10^6^) induced mouse medullary breast adenocarcinoma cells into the right flank of C57BL/6 mice	CA dose: 0–1.25 μg/mL; IV	↓ Tumor volume and weight by ~3.2-fold compared to control	Conjugation of FA with CA-IONPs facilitated active targeting resulting in folate receptor-mediated internalization and enhanced tumor retardation	Shetty et al. [24]

CE, cinnamon extract; CA, cinnamaldehyde; IC50, half maximal inhibitory concentration; EC50, half maximal effective concentration; IV, intravenous; MMP, matrix metallopeptidase; MAPK, mitogen-activated protein kinase, ROS, reactive oxygen species; TCA, trans-cinnamaldehyde; PARP, poly(ADP-ribose) polymerase; NF-κB, Nuclear factor kappa-light-chain-enhancer of activated B cells; DEF-45, DNA fragmentation factor 45; EPR, enhanced permeability and retention; CA-FU NPs, 5-fluorouracil functionalized cinnamaldehyde nanoparticles; BSA-CA NPs, bovine serum albumin–cinnamaldehyde nanoparticles; TCA-CMPs, trans-cinnamaldehyde chitosan microparticles; B-C-CF-CA NPs, Biotin–casein–calcium ferrite–cinnamaldehyde nanoparticles; CA/DATS@PLGA-PEG NPs, cinnamaldehyde–diallyl trisulfide nanoparticles@Polylactic-co-glycolic-polyethylene glycol; HA-CD/FC-CA NPs, cyclodextrin-modified hyaluronic acid conjugate/ferrocene-modified cinnamaldehyde prodrug; MON CA-TPP@HA, mesoporous organosilica nanoparticles–cinnamaldehyde-triphenylphosphine@hyaluronic acid; RSL3@PCA; RAS-selective lethal small molecule 3@acid responsive nanoparticle; DOX-CLC NPs, doxorubicin–lactobionic acid-modified chitosan nanoparticles, ETS@PCA-CA; etoposide@ polycarbonate–cinnamaldehyde; ErbB2, epidermal growth factor receptor 2; HSF1, heat shock protein transcription factor-1; LDHA, lactate dehydrogenase A; PI3k-AKT-mTOR, phosphatidylinositol-3-kinase-mammalian target of rapamycin; COX-2, cyclooxygenase-2; Bcl-2, B-cell leukemia/lymphoma 2 protein; Bax, Bcl-2-associated protein x; VEGF, vascular endothelial growth factor; GSH, glutathione; GPx4, glutathione peroxidase 4; CA-TA-gelled oil NPs, cinnamaldehyde-tannic acid gelled oil NPs; CA-IONPs/FA, iron oxide nanoparticles-loaded CA functionalized with folic acid. ↑ represents increase or upregulation, while ↓ denotes decrease or downregulation.

**Table 3:** Effects of cinnamon extract, cinnamon oil and cinnamaldehyde on obesity, cardiovascular disease and diabetes using in vitro and in vivo models.

Disease Condition	In Vitro/Animal Model	Induction (Mode, Dose)	CE/CO/CA (Mode, Dose, Duration)	Outcome	Reference
Obesity	3T3-L1 cells	Glucose	CA/cinnamyl isobutyrate (30 μM, 12 days)	↓ phospholipid accumulation, triglyceride, PPARγ, C/EBPα, C/EBPβ through involvement of TRPA1	Hoi et al. [117]
	Wistar rats	Early overnutrition-induced metabolic alterations	CA (oral gavage, 40 mg/kg/day, 29 days)	↓ visceral adiposity, serum triglyceride, Srebf1, Acaca, insulin resistance, phospho-eIF2α and IRE1α, endoplasmic reticulum stress and ↑ LC3II/I and Sqstm1.	Neto et al. [118]
	Wistar rats	HFD + sucrose diet	CA (IP, 20 mg/kg/day, 8 weeks)	↓ triglycerides, adiposity index, free fatty acids, total cholesterol, CD11c, CD11b, F4/80, TNF and ↑ Nrf2	Sena et al. [26]
	Wistar rats	HFD-induced atherosclerosis	CA (oral gavage, 20 mg/kg/day, 10 weeks)	↓ total cholesterol, triglycerides, LDL, free fatty acids, creatine kinase, creatine kinase-MB, LDH, AST	Ismail et al. [29]
	Male Swiss mice (8 weeks)	HFD	CE-functionalized Au@P-NPs (oral gavage; 10 mg/kg/day, 8 weeks)	↓ fat deposition, body weight, inflammation, endotoxaemia and ↑ insulin sensitivity, glucose tolerance and UCP1 ↑ *A. muciniphila* and *Bifidobacterium* and ↓ *Lactobacillus*	Sharma et al. [27]
CVD	HUVECs	H_2_O_2_ and TNF-α	CA and methoxyCA (20 µM, 24 h)	↓ oxidative stress and ↑ Nrf2, HO-1 through P38 pathway. Inhibition of monocyte cell adhesion to HUVECs by ↓ VCAM-1.	Kim et al. [28]
	SD rats	LPS (SC, 0.5 mg/kg)	CA (IP, 50 mg/kg, single dose)	↓ inflammatory cell infiltration	Kim et al. [28]
	Leptin receptor deficient diabetic mice (6–8 weeks)	Diabetic db/db mice model	CA (diet containing 0.02% CA, 12 weeks)	↓ ROS, nitrotyrosine, P22, P47, Nrf2, HO-1, quinone oxidoreductase-1 and ↓ p-eNOS accompanied by regulation of NO and improvement of relaxation of aortas and mesenteric arteries	Wang et al. [119]
	Wistar rats	HFD + sucrose diet	CA (IP, 20 mg/kg/day, 8 weeks)	↓ vascular oxidative stress, inflammation, endothelial dysfunction, MDA, 8-OHdG and ↓ Nrf2	Sena et al. [26]
	Primary rat VSMCs, murine macrophage RAW 264.7 and human macrophage THP-1 cells	RAW 264.7 and VSMC cell proliferation in high-glucose DMEM medium and THP-1 cell proliferation in RPMI medium	CA (EC_50_: 131 µM, 24 h) CA pluronic micelles (EC_50_: 84 µM, 24 h)	↓ VSMC proliferation and ↑ Nrf2, SOD and GSH through efficient cellular uptake of CA/CA pluronic micelles with ↓ nitrite and enhanced accumulation on VSMCs	Cartaya et al. [88]
	Neonatal rat cardiomyocytes	Phenylephrine (50 µM)-induced cardiac hypertrophy	TCA (5 µM, 24 h)	↓ phosphorylation and nuclear localization of CaMKII and ERK ↓ hyperphosphorylation of ryanodine receptor type 2 and phospholamban ↑ calcium handling, sarcomere shortening	Qian et al. [120]
	C57BL/J mice (8 weeks)	Phenylephrine (SC, 75 mg/kg/day for 2 weeks)	TCA (oral gavage, 50 and 100 mg/kg/day, 2 weeks)	↓ phosphorylation of CaMKII and ERK through ↓ hypertrophic genes (Nppa, Nppb and Mhy7)	Qian et al. [120]
	Wistar rats	HFD-induced atherosclerosis	CA (oral gavage, 20 mg/kg/day, 10 weeks)	↓ IL-1β, IL-17, IL-6, TNF-α ↑ SOD, CAT, glutathione S-transferase, glutathione peroxidase and ↓ MDA	Ismail et al. [29]
	Mice	Dyslipidemia-induced diet (14 days)	CE (oral gavage, 300, 400 and 500 mg/kg/day, 7 days)	↓ total cholesterol, triglyceride, LDL and ↑ HDL, antiplatelet activity through ↑ bleeding time, coagulation and ↓ ATP-induced platelet aggregation	Sandhiutami et al. [121]
Diabetes	Female virgin albino Wistar rats	FSD/STZ (IP, 25 mg/kg)	CA (oral gavage, 20 mg/kg/day, 15 weeks); glyburide/metformin-HCl (oral gavage, 0.6/100 mg/kg/day, 15 weeks)	↓ maternal and fetal glycemia, placental vasculopathy, fetal hypoxia, redox signaling	Hosni et al. [122]
	3T3-Ll pre-adipocytes	glucose (25 mM) + insulin (1 μM)	CA (100 μg/mL), metformin (10 mM) and CA + metformin (100 μg/mL + 10 mM)	↑ GLUT4 protein, miR26-b ↓ MiR320	Naghiaee et al. [123]
	Male albino rats	60% fructose	CA (oral gavage, 40 mg/kg, 4 weeks); metformin (oral gavage, 300 mg/kg, 4 weeks)	↓ plasma glucose, HbA1c, total cholesterol, LDL cholesterol, triglyceride ↑ GSH, SOD and restored plasma levels of ALT, AST, creatinine, uric acid	Rashwan et al. [124]
	Male albino rats	HFD and STZ (IP, 55 mg/kg)	CA (oral gavage, 40 mg/kg/day, 4 weeks); Metformin (oral gavage, 200 mg/kg/day, 4 weeks); CA + metformin (oral gavage, 40 + 200 mg/kg/day, 4 weeks	↓ blood glucose and improved lipid profile	Ghazal et al. [25]
	Db/db diabetic mice with FBG > 7.0 mM	-	CA (oral gavage, 78/39/19.5 mg/kg, 6 weeks); KP (oral gavage, 116/58/29 mg/kg, 6 weeks); CA + KP (oral gavage, 78 + 116 mg/kg, 39 + 58 mg/kg, 19.5 + 29 mg/kg, 6 weeks)	CA and KP combination ameliorated glucose and lipid metabolism disorders by enhancing lipid metabolism via AMPK activation	Gao et al. [125]
	Wistar Albino rats (7–8 weeks)	STZ (IP, 45 mg/kg)	CA (oral gavage, 20 mg/kg, 30 days)	↓ blood glucose, HbA1c, triglyceride, total cholesterol, VLDL, LDL, urea and ↑ GSH, G6PD	Celik et al. [126]
	Male Wistar rats (6–8 weeks)	HFD and STZ (IP, 35 mg/kg)	CA (oral gavage, 10 mg/kg, 60 days)	↓ ALT, AST, AGEs, aortic RAGE, MDA and ↑ GSH, SOD, mRNA of IRS1, PI3K-P85, AKT2, eNOS, AKT2, IRS1 through IRS1/PI3K/AKT2 pathway	Abdelmageed et al. [127]
	Male C57 mice (6 weeks)	STZ (IP, 150 mg/kg, 3 days)	CA (oral gavage, 20 mg/kg, 7 weeks)	Improved glucose metabolism and insulin sensitivity, ↑ glycogen synthesis and alleviated myocardial injury, ↑ *L. johnsonii* and ↓ *L.murinus* in gut microbiota, ↑ *Faecalibacterium prausnitzii*, IRS1, AKT2, E2F1	Zhao et al. [128]
	Wistar rats (8–12 weeks old)	STZ (IP, 35 mg/kg)	CO nanoemulsion (oral gavage, 6.25/12.5/25 mg/kg, 28 days)	↓ blood glucose, AST, ALT, ALP, triglyceride, pancreatic β-cell damage, and ↑ insulin secretion	Sriramavaratharajan et al. [129]
	Albino Wistar male rats (8-weeks)	STZ (IP, 60 mg/kg)	CE (oral gavage, 500 mg/kg, 28 days), CE + glibenclamide (oral gavage, 300/400/500 mg/kg CE + 3 mg/kg glibenclamide, 28 days	↓ blood glucose and ↑ Fructose-1,6-bis phosphatase	Vijayakumar et al. [130]
	Wistar male rats (6-weeks)	STZ (IP, 230 mg/kg)	CE (oral gavage, 20–60 mg/kg, 4 weeks), CE nanoemulsion (oral gavage, 20–60 mg/kg, 4 weeks)	↓ fasting blood glucose, oral glucose tolerance test value, insulin resistance index, total cholesterol, triglyceride, AST, ALT, creatinine	Huang and Chen [4]
	Adult zebrafish	Glucose (110 mM added to tap water for 2 weeks)	GA-COOE-SeNPs (oral gavage, 10/20 µg/L, 14 days)	↓ blood glucose, ROS, cholesterol, triglycerides and ↑ antioxidant, antilipidemic and hypoglycemic effects	Gutierrez et al. [59]
	BMSCs, RAW 264.7 cells and bacterial cells of *S. mutans* and *P. gingivalis*	Glucose (22 mM glucose + 1 μg/mL PG-LPS)	TNT-CA (7 days)	Acid responsive TNT-CA exerted osteogenic, anti-inflammatory and antimicrobial effects.	Lee et al. [131]

CE, cinnamon extract; CA, cinnamaldehyde; CVD, cardiovascular disease; IP, intraperitoneal; SC, subcutaneous; STZ, streptozotocin; HFD, high-fat diet; FSD, fat sucrose diet; PG-LPS, *Porphyromonas gingivalis* lipopolysaccharide; AMPK, adenosine monophosphate protein kinase; GSH, glutathione; p-eNOS, phospho-endothelial nitric oxide synthase; AKT2, serine/threonine kinase 2; IRS1, insulin receptor substrate 1; NOX4, nicotinamide adenine dinucleotide phosphate hydrogen (NADPH) oxidase 4; TNF-α, tumor necrosis factor-alpha; CD11b and CD11c, a biomarker of macrophages and microglia; phospho-eIF2α, phosphorylated α subunit of eukaryotic initiation factor 2; IRE1α, Inositol-requiring transmembrane kinase/endoribonuclease 1α; PPARγ, peroxisome proliferator-activated receptor; C/EBPα and C/EBPβ, cytosine–cytosine–adenosine–adenosine–thymidine (CCAAT)-enhancer-binding proteins; UCP1, uncoupling protein 1 of mitochondria; IL, interleukin; GLUT4, glucose transporter type 4 protein; Srebf1, sterol regulatory element-binding transcription factor 1; Acaca, acetyl-CoA carboxylase alpha; Sqstm1, sequestosome-1; LC3II/I, light chain 3-phosphatidylethanolamine conjugate; Nrf2, nuclear factor erythroid 2–related factor 2; creatine kinase-MB, creatine kinase-myocardial band; LDH, lactate dehydrogenase; AST, aspartate aminotransferase; ALT, alanine aminotransferase, HO-1, heme oxygenase-1; ROS, reactive oxygen species; HUVECs, human umbilical vein endothelial cells; VCAM-1, vascular cell adhesion molecule 1; NO, nitric oxide; MDA, malondialdehyde; 8-OHdG, 8-hydroxy-2-deoxyguanosine; VSMC, vascular smooth muscle cells; SOD, superoxide dismutase; CaMKII, calcium/calmodulin-dependent protein kinase II; ERK, extracellular signal-related kinase; TNT-CA, cinnamaldehyde functionalized titanium dioxide nanotubes; LDL, low-density lipoprotein; VLDL, very-low-density lipoprotein; HDL, high-density lipoprotein; ATP, adenosine triphosphate; Nppa, natriuretic peptide A; Nppb, natriuretic peptide B; Mhy7, myosin heavy chain 7; GA-COOE-SeNPs, gum Arabic-stabilized *Cinnamomum verum*/*Origanum majorana*/*Origanum vulgare* extracts-based selenium nanoparticles. ↑ represents increase or upregulation, while ↓ denotes decrease or downregulation.

**Table 4:** Effects of cinnamon extract, cinnamon oil and cinnamaldehyde on various neurological disorders in both in vitro and animal models.

Disease Condition	In Vitro/Animal Model	Induction (Mode, Dose)	CE/CA (Route, Dose, Duration)	Outcome	Reference
Alzheimer’s	Male Wistar rats	STZ (ICV, 3 mg/kg) Insulin (ICV, 3 mU/day, 2 weeks)	CA (IP, 10, 100 mg/kg/day, 2 weeks)	Improved recognition/spatial memory deficits and anxiety. ↑ Phospho.GSK-3βSer9/Total. GSK-3β, Phospho.AKTSer473/Total AKT ratios and ↓ Phospho. IRS-1Ser307/Total. IRS1 ratio	Bagheri-Mohammadi et al. [30]
	SD rats	STZ (IP, 4 mg/kg) Insulin (5 mU/day, 2 weeks)	CE (oral gavage, 200 mg/kg, 2 weeks)	↑ GLUT1, 3, and 4 genes in the hippocampal tissue protein. Improved rat performance in the Morris water maze test and behavioral test	Sajadi et al. [140]
	Female adult Wistar rats	STZ (IP, 30 mg/kg)	TCA (oral gavage, 60 mg/kg, 4 weeks)	↓ Astrogliosis, pyknosis, neurodegenerative changes in the hippocampus	Olorunnado et al. [141]
	Male adult Wistar rats (6 weeks)	AlCl_3_ (IP, 100 mg/kg	CE (oral gavage, 200 mg/kg/day, 60 days)	↓ Aβ deposition, neurofibrillary degeneration, neuritic plaques and ↑ number of purkinje cells and dendritic arborization density, perineuronal space, dendritic spines, memory and intellectual performance	Mustafa [142]
	Alzheimer 5XFAD mice model (5 months old)	-	TCA (IP, 30 mg/kg, 8 weeks)	↓ Aβ deposition, β-secretase, cognitive impairment and ↑ SIRT1, PPARγ, PGC1α alleviating AD pathology via SIRT1-PGC1α-PPAR pathway	Do et al. [143]
	Male NMRI mice (50–60 days)	Scopolamine (IP, 1 mg/kg)	CA (oral gavage, 100 mg/kg, 10 days)	Restored amnesia and hippocampal AKT and MAPK dysregulation effect of scopolamine	Kazerouni et al. [144]
Parkinson disease	PC12 cells	6-OHDA (100 μM)	CE (2.5–20 μg/mL, 24 h) CA (1.25–10 μM, 24 h)	↑ cell viability, survivin and ↓ ROS, cytochrome-C, phospho-p44/42, p44/42 resulting in protective action against 6-OHDA-induced cytotoxicity	Ramazani et al. [145]
	SH-SY5Y cells	MPP^+^ (1 mM)	CE (5–50 μM, 12 h)	↑ cell viability, p-ERK1/2, t-ERK1/2 and ↓ ROS, Bcl-2/Bax ratio resulting in neuroprotection against MPP^+^-induced cytotoxicity	Xu et al. [146]
	Male SD rats (6 weeks)	IP; rotenone-induced group (2 mg/kg)	CE nanoemulsion (oral gavage, 60 mg/kg, 5 weeks)	↑ dopamine, tyrosine hydroxylase, CAT, SOD, GSH peroxidase and ↓ α-synuclein, malondialdehyde	Wang et al. [5]
Ischemic stroke	C57BL/6JNarl mice	Ischemia/Reperfusion surgery for I/R brain injury	CA (oral gavage, 10–30 mg/kg)	↓ Infarct area in I/R-induced brain damage, neurological deficit score, COX-2 protein, neuron apoptosis, NR2B, cytochrome-C, caspase-9, caspase-3	Chen et al. [147]
	Male Sprague–Dawley (SD) rats	IP; Middle cerebral artery occlusion model	CE + *Angelica sinensis* extract (oral gavage, 1.6, 3.2, 6.4 g/kg, 7 days)	↑ Iba1, LC3 II, Beclin-1 CD206 and ↓ TNF-α, IL-1β, IL-6, TLR4, phosphorylated-IKKβ, IκBα, NLRP3, nuclear P65, caspase-1, caspase-8, cleaved caspase-3	Luo et al. [148]
	122 patients (81 men and 41 women)	-	Aspirin–cinnamon group and aspirin–placebo group	↓ blood lipid, blood glucose, inflammation, lipoprotein- related phospholipase A2, carotid atherosclerosis. Aspirin-cinnamon groups exhibited superior effects for reducing 90-day recurrent stroke.	Zhang et al. [149]
Trauma brain injury	Male Wistar albino rats	Traumatic brain injury induced by weight-drop model	CA (IP, 100 mg/kg, single dose)	↓ myeloperoxidase, histologic damage scores, ROS, leukocyte infiltration, neutrophil recruitment, acute hippocampal dysfunction	Bektasoglu et al. [150]
	ICR male mice (6–8 weeks)	Traumatic brain injury induced by weight-drop model	CE (oral gavage, 100 μg/mL, 3 weeks)	↓ memory impairment, neuronal loss in temporal cortex and dentate gyrus	Qubty et al. [151]
Multiple sclerosis	Female SJL/J mice (4–5 weeks)	Incomplete Freund’s adjuvant-induced adoptively transferred encephalomyelitis (SC, 400 µg Bovine myelin basic protein + 60 µg *M. tuberculosis*, single dose)	Cinnamon powder (oral gavage, 50 mg/kg, 17 days)	↑ Foxp3+, Treg/Th2 cells and ↓ Th17/Th1 blocking the progression of encephalomyelitis	Mondal and Pahan [152]
	60 patients (36 women and 24 men) with progressive relapsing multiple sclerosis	-	CE (500 mg/capsule, 4 capsules/day, 8 weeks)	↓ pain, C-reactive protein, IL-6, mRNA levels	Delaviz et al. [153]
Migraine	50 migraine patients	-	Cinnamon powder (600 mg/capsule, 3 capsules/day, 60 days)	↓ waist and hip circumference, headache disability, pain level	Zareie et al. [154]
	50 migraine patients	-	Cinnamon powder (600 mg/capsule, 3 capsules/day, 60 days)	↓ IL-6, NO, frequency, severity	Zareie et al. [155]
Depression	Male BALB/c mice (7–8 weeks)	Forced swimming test	TCA (oral gavage, 50 mg/kg/day, 7 days)	↓ immobility during swimming, 5-HT, Glu/GABA in mice hippocampus, COX-2, CB1, TRPV1	Lin et al. [156]
	70 SPF-grade male Kunming mice	Chronic unpredictable mild stress (CUMS), followed by sucrose preference test, forced swimming test, open field test	CO-S-SME (oral gavage, 40, 100 and 200 mg/kg, 8 weeks)	CO-S-SME effectively improve depression-like behavior by ↑ neurotransmitter levels, ↓ corticosterone, IL-6, TNF-α, IL-1β, Firmicutes/Bacteroidetes, Lactobacillus	Ma et al. [157]

CE, cinnamon extract; CA, cinnamaldehyde; TCA, trans-cinnamaldehyde; IP, intraperitoneal; ICV, intracerebroventricular; STZ, streptozotocin; SD, Sprague–Dawley; LPS, lipopolysaccharide; CAT, catalase; SOD, superoxide dismutase; GSH, glutathione; IL-interleukin; TNF-α, tumor necrosis factor-alpha; COX-2, cyclooxygenase-2; CB1, cannabinoid receptor 1; TRPV1, transient receptor potential vanilloid-1; Glu/GABA, glutamate/γ-aminobutyric acid; Foxp3+ regulatory T cells, forkhead box P3 transcription factor-expressing regulatory T cells; Treg/Th2, regulatory T cells/T helper 2 cells; nuclear P65, nuclear transcription factor P65 protein; TLR4, toll-like receptor 4; IKKβ, inhibitory kappa B kinase beta; NLRP3, nucleotide-binding domain, leucine-rich-containing family, pyrin domain-containing-3 inflammasome; NR2B, N-methyl D-aspartate receptor subtype 2B; I/R, renal ischemia/reperfusion; Iba1, ionized calcium-binding adapter molecule 1; IRS1, insulin receptor substrate 1; CD206, cluster of differentiation 206 of mannose receptor; NO, nitric oxide; 6-OHDA, 6-hydroxydopamine neurotoxin; AKT, serine/threonine kinase; MAPK, mitogen-activated protein kinase; GLUT1, glucose transporter type 1, 3 or 4 protein; GSK-3β, glycogen synthase kinase 3beta; Ser, serine; MPP^+^, 1-methyl-4-phenylpyridinium; ROS, reactive oxygen species; Aβ, amyloid-beta plaques; 5-HT, 5-hydroxytryptamine; PPARγ, Peroxisome proliferator-activated receptor gamma; PGC1α, Peroxisome proliferator-activated receptor-γ coactivator 1-α; Bcl-2, B-cell leukemia/lymphoma 2 protein; Bax, Bcl-2-associated protein x. ↑ represents increase or upregulation, while ↓ denotes decrease or downregulation.

**Table 5:** Effects of cinnamon extract and cinnamaldehyde on the alleviation of osteoarthritis, rheumatoid arthritis and osteoporosis in both in vitro and animal models.

Bone/Joint Disease	In Vitro/In Vivo Model	Induction (Mode, Dose)	CE/CA (Mode, Dose, Duration)	Outcome	Reference
Osteoarthritis	Human OA chondrocytes	LPS (10 μg/mL)	CA (20 and 50 μM, 24 h)	↓ IL-1β, IL-6, TNF-α, MMP-13, ADAMTS-5, NF-κB, p65, IκB-α through NF-κB signaling pathway.	Chen et al. [171]
	Human fibroblast-like synoviocytes	LPS (1 µg/mL)	CA (10, 20 and 50 µM, 24 h)	↓ IL-1β, IL-6, TNF-α through blocking of TLR4/MyD88 pathway.	Chen et al. [93]
	Human knee articular chondrocytes	IL-1β (10 ng/mL)	CA (2, 5 and 10 µg/mL, 24 h)	↓ IL-8, PGE2, TNF-α, MMP-13, iNOS, COX-2, ADAMTS-5, phosphorylation of AKT and PI3K through PI3K/AKT pathway.	Wu et al. [172]
	RAW 264.7 cells and rat cartilage chondrocytes	LPS (500 ng/mL)-induced RAW 264.7 cells or IL-1β (10 ng/mL)-induced rat cartilage chondrocytes.	Treatment with DHJST (300 µg/mL) or TH (100 µg/mL) for 18 h (RAW 264.7 cells) or 6 h (chondrocytes).	↓ NO, PGE2, iNOS, COX-2 in RAW 264.7 cells for both DHJST and TH ↓ iNOS, MMP-13 in chondrocytes for DHJST and PGE2 in TH	Tseng et al. [31]
	Wistar rats	MSI (50 mL of 80 mg/mL on day 0 and 40 mg/mL on day 6 in left ankle) or type II collagenase (4 mg/kg in left knee)	DHJST or TH (oral gavage, 25 and 50 mg/kg BW, 10 days)	↓ hind-limb weight-bearing, paw edema swelling, hot-plate latent pain response in MSI-induced rats and restoration of weight-bearing distribution and low-grade inflammatory cell infiltration and cartilage thinning	Tseng et al. [31]
Rheumatoid arthritis	human fibroblast-like synoviocytes	IL-1β (20 ng/mL)	CA (40, 60 and 80 nM, 24 h)	↓ IL-6, IL-8, TNF-α. Retarding phosphorylation of JAK2 and STAT1/STAT3 signaling pathway without affecting NF-κB	Cheng et al. [173]
	Lewis female rats	bovine type II collagen (SC, 2 mg/mL)	CA (oral gavage, 75 mg/kg/day, 21 days) or MTX (oral gavage, 0.5 mg/kg/3 days, 21 days)	↓ swollen paw volume, joint swelling, bone erosion, arthritis severity and ↓ IL-6 level in rat serum	Cheng et al. [173]
	BALB/c male mice	Complete Freund’s adjuvant (SC, triple-dose of 0.1 mL of 1 mg mycobacterium tuberculosis/mL paraffin oil, 2 days)	CA (IP, 100 mg/kg/day, 3 weeks) or MTX (IP, 0.75 mg/kg/2 days, 3 weeks)	↓ serum rheumatoid factor, arthritis index, paw swelling, cartilage and bone erosion. ↓ TNF-α, IL-1β, IL-6, IL-23, IL-17 and COX-2 through modulation of NF-κB pathway	El-Tanbouly and Abdelrahman [174]
Osteoporosis	Preosteoblast cells MC3T3-E1.	MC3T3-E1 cells were differentiated with osteogenic medium	CA (25, 50 and 100 µM, 6 days for ALP activity and osteoblastic markers as well as 14 days for mineralization assay)	↑ TGF-β, BMP2/4, pSmad2/3, osteocalcin and Runx2	Hong et al. [175]
	Female C57BL/6J mice	Mice induced with ovariectomy by removing bilateral ovaries	CA (oral gavage, 5, 10 and 20 mg/kg, 5 times per week for 10 weeks)	↑ bone indices and ↑ osteocalcin and procollagen type 1 levels in mice serum through BMP/TGFβ/Smad signaling	Hong et al. [175]
	BMSCs isolated from C57B/6J mice	H_2_O_2_	CA (50 and 100 µM, 24 h)	↓ ROS to reduce oxidative stress ↑ OPA1 and ↓ Drp1 to mitigate mitochondrial dysfunction ↑ Nrf2, HO-1, COL1A1, Runx2, OCN	Lin et al. [176]
	Female C57B/6J mice	mice induced with ovariectomy by removing bilateral ovaries	CA (IG, 50 mg/kg/per, 8 weeks)	↓ ROS and ↑ Nrf2, Runx2. Improvement in bone trabecular microstructure by ↑ bone volume/total volume ratio and plate number	Lin et al. [176]
	SD rats	STZ (IP, 60 mg/kg)	CA (oral gavage, 20 and 40 mg/kg/per day, 12 weeks)	↑ bone strength, remodeling activity and structure ↑ bone volume/tissue volume ratio, trabecular plate number thickness ↓ bone surface area/bone volume ratio and trabecular spacing ↑ netrin-1, DCC, UNC5B, RANKL, OPG ↓ TGF-β, cathepsin, TRAP, RANK	Ji et al. [32]

CE, cinnamon extract; CA, cinnamaldehyde; OA, osteoarthritis; LPS, lipopolysaccharide; IL, interleukin; TNF-α, tumor necrosis factor-alpha; NF-κB, nuclear factor kappa-light-chain-enhancer of activated B cells; IκB-α, nuclear factor of kappa light polypeptide gene enhancer in B-cells inhibitor alpha; MMP-13, matrix metallopeptidase-13; PGE2, prostaglandin E2; iNOS, inducible nitric oxide synthase; COX-2, cyclooxygenase-2; ADAMTS-5, a disintegrin and metalloproteinase with thrombospondin motifs 5; AKT, serine/threonine kinase-2; PI3K, phosphatidylinositol 3-kinase; TLR4/MyD88, Toll-like receptor-4/Myeloid differentiation primary response-88; DHJST, Du-Huo-Ji-Sheng-Tang (Chinese herbal formulation with 15 herbs); TH, Qi-tonifying herbs (Chinese herbal formulation with 4 herbs); MSI, monosodium iodoacetate; STAT1, signal transducer and activator of transcription 1; STAT3, signal transducer and activator of transcription 3; ALP, alkaline phosphatase; TGF-β, transforming growth factor-beta; OCN, osteocalcin; BMSCs, bone mesenchymal stromal cells; ROS, reactive oxygen species; OPA1, optic atrophy 1; Drp1, dynamin-related protein 1; Nrf2, nuclear factor erythroid 2–related factor 2; HO-1, heme oxygenase 1; COL1A1, collagen type 1 alpha 1; Runx2, Runt-related transcription factor 2; DCC, netrin receptor deleted in colorectal cancer; UNC5B, uncoordinated-5 netrin receptor; RANK, receptor activator of nuclear factor kappa B ligand; RANKL, RANK ligand; TRAP, tartrate-resistant acid phosphatase; OPG, osteoprotegerin; IP, intraperitoneal; IG, intragastric; SC, subcutaneous; MTX, methotrexate. ↑ represents increase or upregulation, while ↓ denotes decrease or downregulation.
